# Virulence and Antimicrobial Resistance Traits of *Escherichia coli* Retrieved from Fermented Dairy Products During Ramadan in Egypt: Seasonal Public Health Implications

**DOI:** 10.3390/antibiotics15050483

**Published:** 2026-05-09

**Authors:** Fatma Elzhraa, Gabriella Kiskó, Ágnes Belák

**Affiliations:** 1 Department of Food Microbiology, Hygiene and Safety, Institute of Food Science and Technology, Hungarian University of Agriculture and Life Sciences, 1118 Budapest, Hungary; mohamed.fatmaelzhraa.ragab.osman@phd.uni-mate.hubelak.agnes@uni-mate.hu (Á.B.); 2 Department of Food Hygiene, Safety, and Technology, Faculty of Veterinary Medicine, Mansoura University, Mansoura 35516, Egypt

**Keywords:** *E. coli*, fermented dairy food, virulence genes, antimicrobial stewardship, public health hazard

## Abstract

**Background:** Rayeb milk and yogurt provide lasting energy and maintain hydration during fasting. The surge in demand during Ramadan (the holiest month of the year across the Islamic world) increases production to meet consumer needs, potentially compromising strict adherence to safety standards. Data on the prevalence and characterization of *Escherichia coli* (*E. coli*) in fermented dairy products during Ramadan, a model of increased batch turnover, remain to be investigated. **Objectives:** This study aimed to identify the AMR profile and molecularly characterize the AMR and virulence traits of *E. coli* isolates prevalent in fermented milk products. **Methods**: A total of 34 *E. coli* isolates, representing eight distinct serotypes, were recovered from 150 fermented milk samples; rayeb milk (*n* = 75) and yogurt (*n* = 75) collected from densely populated towns in the Dakahlia Governorate, Egypt. **Results**: The most prevalent serotypes were O153:H2, O125:H21, and O119:H6, followed by O111:H2, O26:H11, and O127:H6. The virulence genes *stx1* and *stx2* were detected in 76.47% of isolates, while *eaeA* and *hlyA* were found in O26:H11, O119:H6, and O55:H7 serotypes. In total, 73.53% (25/34) of *E. coli* isolates were classified as MDR, while 26.47% (9/34) exhibited XDR. Resistance was most prevalent against penicillin, tetracycline, ciprofloxacin, ampicillin, kanamycin, chloramphenicol, and trimethoprim. The *kan*, *dfrA*, *blaOXA-1*, *tetA(A)*, and *van(A)* AMR genes were positive in 70.59%, 67.65%, 61.76%, 100%, and 47.06% of isolates, respectively. **Conclusions**: Identified *E. coli* AMR and virulence panels reflect that production pressure can challenge strict adherence to hygiene control measures and cold-chain maintenance. Subsequently, authorities must enforce proper quality assurance protocols to minimize the risk of food poisoning.

## 1. Introduction

Fermented dairy products are widely consumed in the Middle East and North Africa, particularly in Egypt, especially during Ramadan, a month in which healthy adolescents and adults are obligated to fast daily from dawn to sunset and consume only two main meals (Iftar after sunset and Suhoor before dawn) [1]. This shift in meal frequency and timing considerably alters dietary patterns, increasing reliance on nutrient-dense, easily digestible foods, especially fermented dairy products such as yogurt and rayeb milk [2]. Yogurt is produced through lactic acid bacteria (LAB)-based fermentation of milk using starter cultures such as *Streptococcus thermophilus* and *Lactobacillus delbrueckii* subsp. *bulgaricus*, and other probiotic strains to enhance its tangy flavor, nutritional, and health benefits [3]. Traditional rayeb milk is fermented raw or heat-treated milk, with fermentation driven by indigenous lactic acid bacteria (*Lactococcus lactis*, *Lactococcus lactis* subsp. *lactis, Lactobacillus delbrueckii* subsp. *bulgaricus*, *Lactobacillus acidophilus*, *Leuconostoc mesenteroides*, and *Streptococcus thermophilus*) under ambient conditions, giving the product an acidic flavor and a semisolid texture [4]. Cultured dairy products retain probiotic effects and a bioavailable nutritional profile of high-quality protein, calcium, B vitamins, phosphorus, and other essential micronutrients, making them an essential part of a meal to support hydration and gut health during prolonged fasting (~14–16 h) [5,6]. Adding commercial starter culture (*Streptococcus thermophilus*, *Lactobacillus acidophilus*, *Bifidobacterium* spp.) to standardize the fermentation process of the industrial rayeb milk confers probiotic and health-promoting benefits through the production of organic acids (lactic and acetic acids) and antimicrobial substances (bacteriocins) in the intestinal medium to limit pathogen colonization and modulate gut microbiota [7].

Indeed, the dense consumer markets and overrated demand for fermented milk products during Ramadan put pressure on limited suppliers to meet consumer demand, often at the expense of hygiene and safety control in dairy processing, thereby increasing the risk of contamination by microbial hazards [8,9]. A review of the literature from low-income, highly populated countries has reported that the widespread presence of small-scale informal producers, a lack of incentives, inadequate cold chains, and limited regulatory oversight endanger hygiene standards and facilitate microbial contamination of food [10]. Dairy-based fermented foods made in a hasty manner using unpasteurized milk can act as transmission vehicles, and even with pasteurization, post-process contamination may allow the pathogen to persist through manufacturing, creating serious public health risks [11]. The presence of *Escherichia coli* (*E. coli*) and other potential pathogenic microorganisms in fermented foods may pose a significant food safety risk. Although fermentation processes generally inhibit the growth of pathogenic microbiota, under certain conditions, some strains may persist. Of particular importance are pathogenic variants such as Shiga toxin-producing *E. coli* (STEC), which can cause serious, even life-threatening diseases [12,13]. Infections caused by STEC can lead to a range of clinical manifestations, from asymptomatic cases and mild diarrhea to more serious manifestations such as bloody diarrhea, hemolytic uremic syndrome (HUS), thrombocytopenia, and acute renal failure [13]. Alterations in gastric acidity, mucosal barrier integrity, and immune responsiveness following prolonged fasting influence host susceptibility to more severe clinical manifestations after consuming ready-to-eat products containing foodborne pathogens [14]. While STEC O157 is most commonly linked to outbreaks of bloody and non-bloody diarrhea and HUS, several non-O157 serotypes (O26, O103, O111, O145, O45, O91, O113, O121, and O128) have also been associated with severe foodborne illnesses and gut health impairments [15,16].

Virulent and antimicrobial-resistant (AMR) pathogens pose a global health threat and are projected to cause 10 million deaths each year by 2050 [17]. Another intriguing facet of STEC pathogenicity is primarily linked to specific virulence determinants, including the intimin protein (encoded by the *eae* gene), Shiga toxins (encoded by *stx1* and *stx2*), and the plasmid-encoded alpha (α)-hemolysin (*hlyA*) [18,19,20]. These are often carried on mobile genetic elements such as plasmids, bacteriophages, transposons, and pathogenicity islands, allowing for their horizontal transfer and recombination, and the emergence of hybrid strains with new virulence panels (pathotypes) that may exhibit enhanced pathogenic potential [21].

From the One Health perspective, *E. coli* has been frequently studied as a central model and surveillance organism in AMR research worldwide because of its widespread presence in humans, animals, food sources, plants, and wildlife, making it an excellent sentinel organism to monitor AMR trends and mechanisms across sectors [22]. Growing concerns have arisen about antimicrobial resistance determinants in animal husbandry, particularly the emergence of β-lactamases. *E. coli* acts as a major reservoir of extended-spectrum β-lactamases (ESBLs) and can transfer them horizontally to other commensal or pathogenic Enterobacterales via plasmids, enabling them to hydrolyze third-generation cephalosporins and amplify antimicrobial resistance dissemination [23]. Various multidrug-resistant (MDR) *E. coli* strains recovered from infected dairy animals were more difficult to treat with commonly used antibiotic classes, underscoring the direct impact of resistance on therapeutic success and the need for last-resort antimicrobials [24]. Ready-to-consume dairy products represent a critical interface between animals, food products, and humans, serving as a potential pathway for the transmission of virulent and AMR pathogens to consumers. The incidence of ESBLs-harboring *E. coli* in fermented milk products raises public health concerns about the One Health dimension of community in conjunction with hospital AMR burdens [25].

Egypt, located in the Middle East, is within a region with the highest reported annual incidence of human STEC infections, reaching 152.6 cases per 100,000 people per year, including 160 cases of HUS [26]. Several studies have examined the occurrence of potentially pathogenic virulent and AMR *E. coli* in milk and dairy products across samples collected randomly from Egyptian markets throughout the year. However, investigations specifically targeting fermented dairy products with consideration of seasonal or culturally driven consumption patterns are limited [27,28,29,30,31]. This highlights the importance of strict antimicrobial stewardship and continuous surveillance of virulent and AMR *E. coli* serogroups in dairy products, particularly fermented ones, to improve safety and hygiene practices in dairy processing and handling. In this study, we address these knowledge gaps with an emphasis on STEC prevalence in fermented milk products such as yogurt and rayeb milk, as well as the virulence and AMR determinants of the isolates during Ramadan, which serves as an ideal model of seasonally driven increased demand for fermented foods where intensified production compromises strict adherence to food safety practices and favors the emergence of foodborne pathogens.

## 2. Results

### 2.1. Geographic Mapping of Product Type and City-Location-Influenced E. coli Isolation

**Figure 1:**
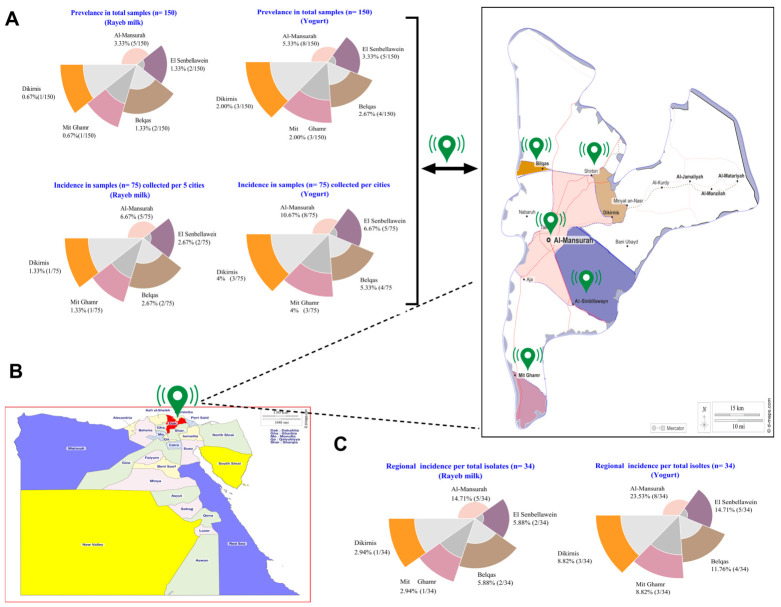
Doughnut charts (**A**,**C**) and geographic map (**B**) illustrate the regional distribution of *E. coli* isolates (*n* = 34) recovered from rayeb milk (*n* = 75) and yogurt samples (*n* = 75).

Out of the 150 investigated rayeb milk and yogurt samples (*n* = 75 each), a total of 34 *E. coli* isolates were confirmed at the species level by MALDI-TOF MS (score ≥ 2.00), corresponding to a total prevalence rate of 22.67% (34/150). Regarding the total samples (*n* = 150), the prevalence was highest for Mansoura rayeb milk (3.33%, 5/150) and Mansoura yogurt (5.33%, 8/150), and was lowest in Mit Ghamr and Dikrinis for both dairy products (Figure 1A). Compared with rayeb milk, from which only 11 *E. coli* isolates (11/75, 14.67%) were recovered, most isolates were obtained from yogurt samples (23/75; 30.67%) in the same surveyed regions, indicating that yogurt is a more hazardous source of *E. coli* among the local dairy products studied (Figure 1A).

Concerning the total number of *E. coli* recoveries, yogurt accounted for 67.65% (23/34) of the isolates (Figure 1C), while rayeb milk accounted for 32.35% (11/34). In regard to regional distribution, Mansoura showed the highest incidence rate in both fermented dairy products, with 38.23% (13/34) of the isolates, followed by El Senbellawein (7/34; 20.6%) and Belqas (6/34; 17.6%), while Dikirnis and Mit Ghamr provided an equal incidence rate, reaching 11.8% (4/34).

### 2.2. Incidence of Serogroups and Virulent Genes Across the Isolated E. coli Strains

Serotyping of *E. coli* isolates based on visible agglutination with O and H antigens identified eight distinct serovars among 34 isolates recovered from rayeb (*n* = 11) and yogurt (*n* = 23) samples (Table 1). In total, the most prevalent serotypes were O153:H2 (7/34, 20.59%), O125:H21 and O119:H6 (each 5/34, 14.7%), followed by O111:H2, O26:H11 and O127:H6 (each 4/34, 11.7%). Other frequent serotypes included O103:H2 (3/34, 8.8%) and O55:H7 (5.9%, 2/34) among all isolates. In rayeb, the most prevalent serotypes were O26:H11 (4/11, 36.4%), followed by O153:H2 and O127:H6 (3/11, 27.3%), and a single O119:H6 isolate (1/34, 2.9%). In yogurt, the O125:H21 was the most frequent serogroup (5/23, 21.7%), followed by O111:H2, O119:H6, and O153:H2 (each 4/23, 17.4%), O103:H2 (3/23, 13.0%), O55:H7 (2/23, 5.8%), and O127:H6 (1/23, 4.3%). The distribution of identified serovars was not statistically different (*p* > 0.05) between the two fermented dairy products, except for O26:H11, which was detected only in rayeb samples.

**Figure 2:**
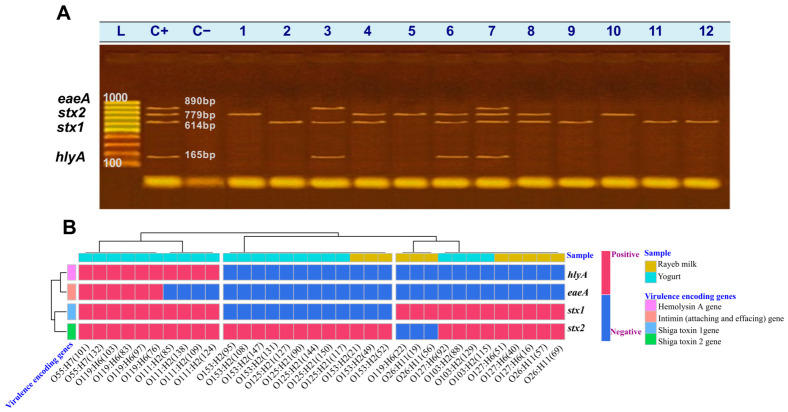
(**A**) Representative gel electrophoresis showing the multiplex-PCR amplified *eaeA* (890 bp), *stx2* (779 bp), *stx1* (614 bp), and *hlyA* (165 bp) virulence genes of *E. coli* isolated from rayeb milk (*n* =11) and yogurt (*n* =23). Lanes: L: DNA ladder (100 bp), C+: positive control (*E. coli* O157:H7, ATCC 43895), C−: No template control. 1&10: strains with serotype O153:H2, 2&8: strains with serotype O127:H6, 3: O55:H7, 4: O103:H2, 5: O125:H21, 6: O111:H2, 7&11: strains with serotype O119:H6, and 9&12: strains with serotype O26:H11; (**B**) a heatmap with hierarchical Ward’s clustering based on Euclidean distance visualizes the incidence and diversity of virulence gene markers across all *E. coli* isolates (*n* = 34). The *X*-axis shows *E. coli* serotypes, ID numbers, and isolation samples. The *Y*-axis shows the tested virulence genes. Red cells indicate gene existence; light blue cells indicate gene absence.

PCR screening for virulence genes confirmed a high incidence rate of Shiga toxin genes (*stx1* and *stx2*), each detected in 26/34 isolates (76.5%), followed by *hlyA* gene (11/34 isolates, 32.4%) and *eaeA* gene (6/34 isolates, 17.6%), highlighting the high virulence potential and heterogeneous genetic makeup of *E. coli* across different serotypes and dairy products (Figure 2A,B). Yogurt-retrieved O119:H6 isolates exclusively harbored the complete virulence gene set (*eaeA*, *stx1*, *stx2*, *hlyA*), whilst O111:H2 and O55:H7 serotypes carried different combinations of three virulence genes. The O103:H2, O125:H21, O153:H2, and O127:H6 serotypes carried at least one of the Shiga toxin genes (*stx1* and *stx2*). Regarding rayeb milk, O127:H6 isolates exhibited co-occurrence of *stx1* and *stx2*, whereas other isolates consistently carried only one Shiga toxin gene. Of note, the *hlyA* marker was identified only in the O119:H6, O111:H2, and O55:H7 isolates retrieved from yogurt. Different genes were also detected in isolates of the same serotype, as observed for O26:H11.

Notably, more than half of the isolates (55.88%) harbored two or more targeted virulence genes, indicating enhanced pathogenic potential for *E. coli* recovered from fermented dairy foods. Virulence gene co-occurrence patterns across the 34 *E. coli* isolates showed a high incidence of *stx2* alone in 12 isolates (35.29%), followed by *stx1* + *stx2* in 9 isolates (26.47%). A smaller proportion harbored *stx1* alone (3 isolates, 8.82%). Complex combinations of a full virulence gene profile (*stx1* + *stx2* + *hlyA* + *eaeA*) were observed in 6 isolates (17.65%), while the co-occurrence of *stx1* + *stx2* + *hlyA* was evident in 4 isolates (11.76%). As such, the coexistence of Shiga toxin-encoding genes (*stx1* and/or *stx2*) with adherence (*eaeA*) and hemolysin (*hlyA*) genes reflects the ability of isolated serogroups to cause serious gastrointestinal manifestations.

### 2.3. Susceptibility to Antimicrobial Agents

**Figure 3:**
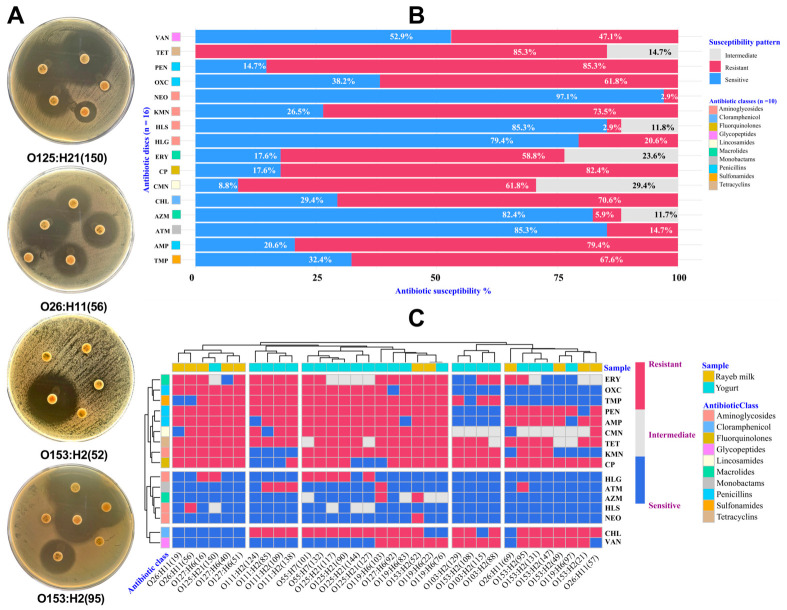
(**A**) Disk-diffusion (Kirby–Bauer) method on Mueller–Hinton agar showing inhibition zones diameter as a standardized approach of antimicrobial susceptibility interpretation using CLSI breakpoints; (**B**) the stacked bar plot shows the cumulative susceptibility of *E. coli* isolates (*n* = 34) to 16 antibiotics across 10 distinct categories; (**C**) a heatmap with hierarchical Ward’s clustering based on Euclidean distance visualizes the AMR profile of *E. coli* isolates (*n* = 34) belonging to eight serogroups. The *X*-axis shows individual *E. coli* serovars, their ID numbers, and the isolation product. The *Y*-axis represents a panel of tested antibiotic discs. Red cell indicates complete resistance; gray cell indicates intermediate susceptibility; blue cell indicates complete sensitivity. PEN, penicillin; OXC, oxacillin; TET, tetracycline; CP, ciprofloxacin; CMN, clindamycin; ERY, erythromycin; AMP, ampicillin; KMN, kanamycin; CHL, chloramphenicol; TMP, trimethoprim; NEO, neomycin; ATM, aztreonam; HLS, high-level streptomycin; AZM, azithromycin; HLG, high-level gentamicin; VAN, vancomycin.

The cumulative susceptibility data of *E. coli* isolates against a profile of 16 antibiotics representing 10 different antibiotic categories revealed a heterogeneous AMR pattern (Figure 3A,B). Resistance was most prevalent against penicillin (PEN) (85.29%), tetracycline (TET) (85.29%), ciprofloxacin (CP) (82.35%), ampicillin (AMP) (79.41%), kanamycin (KMN) (73.53%), chloramphenicol (CHL) (70.59%), and trimethoprim (TMP) (67.65%). Intermediate resistance was notable for clindamycin (CMN) (29.41%) and erythromycin (ERY) (23.53%), suggesting possible emergence of reduced sensitivity. In stark contrast, the highest susceptibility was observed for neomycin (NEO) (97.1%), aztreonam (ATM) (85.29%), high-load streptomycin (HLS) (85.29%), azithromycin (AZM) (82.35%), and high-load gentamicin (HLG) (79.41%), underscoring their relative effectiveness.

The heatmap (Figure 3C) highlights notable variability in AMR patterns and the predominance of β-lactam and TET resistance across a total of 34 *E. coli* isolates belonging to eight serovars. Aminoglycosides (NEO, HLS) and AZM were the most effective agents against rayeb milk and yogurt-derived *E. coli* isolates in vitro. Serotypes O153:H2 (52), O26:H11 (56), O119:H6 (76), O119:H6 (83), O127:H6 (92), O153:H2 (95), O119:H6 (103), O55:H7 (132), and O111:H2 (138) demonstrated the broadest extensive drug resistance (XDR) pattern, with consistent non-susceptibility to β-lactams [PEN, AMP, oxacillin (OXC)] and TET, accompanied by frequent resistance to CP and CHL. Of note, all other serotypes exhibited intermediate MDR profiles, characterized by resistance to β-lactams and TET but partial susceptibility to aminoglycosides (HLG, HLS, KMN, and NEO), and macrolides (AZM and ERY). Both isolates of the O55:H7 serotype were also resistant to most β-lactams yet retained sensitivity to NEO, HLS, and AZM. The most pronounced phenotypic AMR profile was observed in serotype O153:H2 (52) isolated from rayeb milk, which showed resistance to 13 antibiotics, including exclusive resistance to NEO. In contrast, the yogurt-derived O119:H6 (97) isolate exhibited the lowest AMR burden, characterized by susceptibility to most tested antibiotics, with occasionally restricted resistance to PEN, CP, CHL, and vancomycin (VAN).

**Figure 4:**
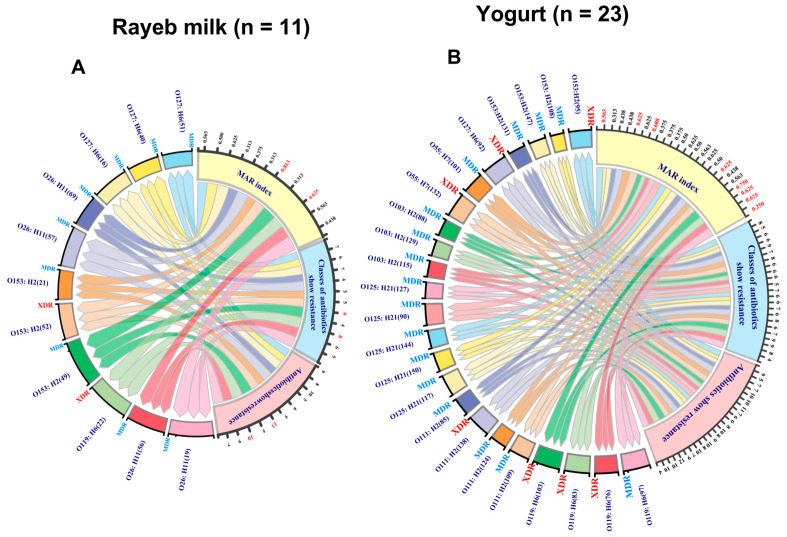
A chord plot visually illustrating complex inter-relationships within *E. coli* isolates (*n* = 34). Arcs with colored nodes show the relationships between antibiotics to which resistance exists, antimicrobial categories in which resistance exists, resistance degrees, and the MAR index in *E. coli* serotypes retrieved from (**A**) rayeb milk (*n* = 11) and (**B**) yogurt (*n* = 23) samples. Features are outside the left serotypes nodes. Each link represents a serotype. Labeling the outside right nodes intuitively reflects the results for each serotype. MAR, multiple antibiotic resistance index; MDR, multidrug-resistant; XDR, extensively drug-resistant.

The results in Figure 4A,B and Table S1 show that 73.53% (25/34) of the *E. coli* isolates were non-susceptible to at least one antimicrobial agent across ≥3 different antimicrobial classes and were labeled MDR. In comparison, 26.47% (9/34) of isolates showed resistance to at least 1 drug in ≥8 tested antimicrobial categories (8/10, 0.8) and were classified as XDR.

Indeed, the majority of *E. coli* strains recovered from rayeb milk (9/11, 81.82%) reached a multiple antibiotic resistance (MAR) index ranging from 0.313 to 0.625, and demonstrated an MDR pattern after displaying susceptibility to only one or two categories and resistance to 5–10 antibiotics (Figure 4A). Of note, O119: H6 (22) and O153:H2 (52) obtained from rayeb milk (2/11, 18.18%) were labeled XDR due to their susceptibility to at least one antimicrobial class, and reached 0.625 and 0.813 MAR index values, respectively, signifying that these isolates are of particular concern, as they reflect contamination of the examined dairy products (Figure 4A).

Notably, seven (7/23, 30.43%) *E. coli* isolates [O153:H2 (95), O153:H2 (131), O55: H7 (101), O111:H2 (85), O111:H2 (109), O119:H6 (103), and O119:H6 (76)] obtained from yogurt samples were labeled XDR and reached > 0.2 (0.56 − 0.75) of the MAR index, signifying that these isolates have emerged from high-risk contamination (Figure 4B). In addition, the remaining sixteen (16/23, 69.57%) *E. coli* isolates retrieved from yogurt showed resistance to 4–9 antibiotics, with MAR index values ranging from 0.25 to 0.625, and were identified as MDR isolates (Table S1).

### 2.4. The Occurrence of AMR Gene Determinants in E. coli Serovars

Serotype-wise clustering revealed that multidrug resistance profiles were particularly prominent among O119:H6, O127:H6, and O153:H2 isolates, suggesting these serotypes may serve as reservoirs of diverse resistance determinants. In support of these suggestions is the widespread presence of clinically important AMR genes in dairy-derived *E. coli*, with yogurt isolates demonstrating a comparatively higher prevalence.

**Figure 5:**
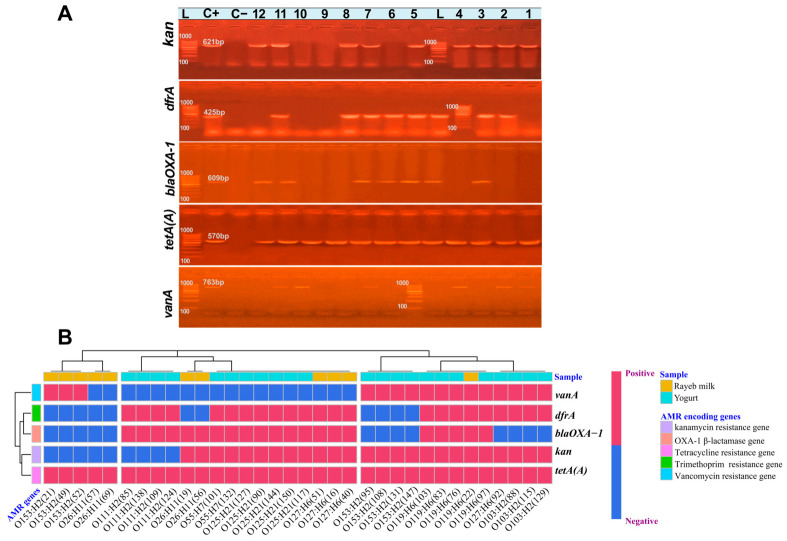
(**A**) Representative gels of electrophoresis visualizing the uniplex-PCR amplified *kan* (621 bp), *dfrA* (425 bp), *blaOXA*-*1* (609 bp), *tetA(A)* (570 bp), *vanA* (763 bp) AMR genes of *E. coli* isolates (*n* = 34). Lanes: L: DNA ladder (100 bp), C+: known PCR-confirmed *E. coli* isolates (positive controls for *blaOXA-1*, *tetA(A)*, *dfrA*, and *kan* genes); *Enterococcus faecalis* ATCC 51299 (positive control for *vanA*), C−: no template control. 1&10: strains of serotype O153:H2, 2&8: strains of serotype O127:H6, 3: O55:H7, 4: O103:H2, 5: O125:H21, 6: O111: H2, 7&11: strains of serotype O119:H6, and 9&12: strains of serotype O26:H11; (**B**) a heatmap with hierarchical Ward’s clustering based on Euclidean distance visualizes the incidence and diversity of AMR gene markers across all *E. coli* isolates (*n* = 34). The X-axis shows *E. coli* serotypes, ID numbers, and isolation samples. The *Y*-axis displays the PCR-amplified AMR genes. Red cells indicate gene existence; light blue cells indicate gene absence.

Gel electrophoresis of U-PCR-targeted AMR genes (Figure 5A) showed clear visualization of *kan*, *dfrA*, *blaOXA-1*, *tetA(A)*, and *vanA* bands among *E. coli* isolates (*n* = 34). It was confirmed that 100% (34/34) of isolates were positive for at least one of the genes investigated. The heatmap (Figure 5B) illustrates the heterogeneous distribution of AMR genes across serotypes and fermented dairy products. All isolates harbored *tetA(A)* (34/34; 100%), while *kan* (25/34; 73.53%) was the most frequently prevalent gene, followed by *dfrA* (23/34; 67.65%), and *blaOXA-1* (21/34; 61.76%), whereas *vanA* was less common (16/34; 47.05%). Yogurt isolates (*n* = 23) carried a higher burden of AMR determinants, with *tetA(A)* (23/23; 100%), *kan* and *dfrA* (19/23; 82.6%), *blaOXA-1* (15/23; 65.22%), and *vanA* (12/23; 52.17%). Rayeb milk isolates (*n* = 11) displayed lower, yet notable, prevalence of *tetA(A)* (11/11; 100%), *kan* and *blaOXA-1* (6/11; 54.55%), *dfrA* and *vanA* (4/11; 36.36%).

A single or multiple AMR gene co-occurrences varied across eight *E. coli* serogroups. The O119:H6 serotypes isolated either from rayeb milk or yogurt samples were labeled XDR and harbored a full set of tested AMR determinants (*blaOXA-1* + *kan* + *dfrA* + *tetA(A)* + *vanA*), accounting for nearly 15% (5/34) of the resistant profiles. The rayeb-milk-recovered MDR O127:H6 isolates, and yogurt-derived O55:H7 exhibited *blaOXA-1* + *kan* + *dfrA + tetA(A)* combinations, while the yogurt-retrieved single XDR O127:H6 and three MDR O103:H2 isolates displayed *kan* + *dfrA* + *tetA(A)* + *vanA* resistance pattern. Yogurt-recovered MDR, and XDR O111:H2 isolates displayed co-occurrence of *blaOXA-1* + *dfrA* + *tetA(A)* AMR gene determinants. Two O26:H11 (isolates No. 19 & 56) harbored three AMR genes (*blaOXA-1* + *kan* + *tetA(A)*). Yogurt-recovered MDR and XDR O153:H2 isolates frequently carried a combination of *kan* + *tetA(A)* + *vanA*. On the other hand, rayeb-milk-recovered MDR O153:H2 and O26:H11 (isolates No. 57 & 69) harbored fewer gene combinations, with *tetA(A)* alone or *tetA(A)* + *vanA* being the dominant combinations.

## 3. Discussion

During Ramadan, there is an increase in the consumption of dairy products, particularly yogurt and fermented milk. These are consumed by Muslims at dawn (Suhoor) for hydration and at Iftar for their nutritional value and their significant impact on GIT function and health [32]. This surge has the potential to exert pressure on safety controls and overwhelm supply chains and sanitation systems, thereby increasing the risk of contamination [33]. Environmental and socioeconomic factors, such as Egypt’s hot climate, the lack of refrigeration for milk, long transport times, and the predominance of smallholder farms, create ideal conditions for such contamination [34]. It is frequently observed that small-scale farmers in the Delta region (Dakahlia governorate) lack proper hygienic milking practices and adequate cooling facilities [35]. These limitations can lead to bacterial proliferation during the milk’s transit phase. In the current work, the elevated incidence of *E. coli* across surveyed yogurt samples (30.7%, 23/75) compared with the rayeb ones (14.7%, 11/75) suggests that yogurt production may entail a heightened microbial risk. The causality of this is unclear, but it could be related to inadequate handling practices and the absence of pasteurization or stringent temperature control in small-scale fermentation processes. When compared with a recent study conducted outside the Ramadan season in Dakahlia Governorate, Egypt [36], lower detection rates of *E. coli* were reported in the investigated yogurt (22%, 11/50) and rayeb milk (4%, 2/50) samples under routine production conditions. This seasonal difference may reflect increased demand during peak consumption periods, which can strain hygienic practices and quality control measures. Nevertheless, in the absence of a direct seasonal comparison within the same study design, this interpretation should be considered cautiously.

It is hypothesized that, given Mansoura’s (the capital of Dakahlia) prevailing market practices, substantial population, and extensive hospital and student accommodation infrastructure, yogurt production and distribution will be considerable even without strict adherence to hygiene control measures and cold-chain maintenance. This trend is particularly pronounced during Ramadan, when yogurt and rayeb milk sales surge [37]. Through recent surveys conducted by our lab members [18], and Ibrahim et al. [38] in Mansoura City, researchers have elucidated the presence of *E. coli* in commercial yogurt and cheese, thereby confirming the potential for fermented dairy products to serve as vehicles for the propagation of pathogenic *E. coli*. In agreement with this observation, the highest number of isolates (35.3% of all positives) was found in Mansoura, reflecting that its dense population and the surge in dairy consumption challenge the strict application of hygiene control measures, thereby increasing the potential risk of microbial contamination or the dissemination of foodborne pathogens. In parallel with the findings of Ibrahim et al. [38], 28% of dairy samples from Dakahlia were contaminated with *E. coli*, with raw market milk (52%) and local cheeses (48%) exhibiting particularly high levels of contamination. In contrast, large-scale industrial yogurt (8% contaminated) was found to be significantly safer, thereby emphasizing the heightened risk associated with informal dairy products, such as those available in Ramadan markets in the current research. Unfortunately, little is currently known about the extent of the interplay among product type (e.g., yogurt > rayeb), environmental heat, high consumption, and socioeconomic conditions. A correlation may be observed between the number of recoveries in highly populated cities of the Delta region (Mansoura, El Senbellawein, Belqas, Dekernes, Mit Ghamr) and local production scale and market size. In the search for the given Dakahlia’s climatic conditions and prevailing market practices [39], a substantial post-harvest bacterial load often remains unclarified.

Intriguingly, the dominant serogroups were a diverse set of non-O157 serovars, including O153:H2, O125:H21, O119:H6, O111:H2, and O26:H11. These serogroups are closely aligned with the previous survey in Mansoura city by Ibrahim et al. [38], who found that 8% of large-scale yogurt and rayeb milk were positive for the O111:H2, O117:H4, O91:H21, and O86 serogroups. Small-scale yogurt showed a 25% prevalence of O125:H21 and O55:H7 serogroups in Beni Suef Governorate, which is geographically adjacent to Dakahlia [29]. Furthermore, the O55:H7, O127:H6, and O119:H6 serogroups were reported in raw milk, cheeses, and ice cream obtained from supermarkets, shops, and local groceries across Egypt by our lab member Elafify et al. [28]. Of particular significance in the current research was the identification of O26:H11 and O111:H2 in ready-to-consume fermented milk products, which are epidemiologically and pathotypically related to O157:H7. In accordance with that, Elmonir et al. [26] reported O26 (4.5%) and O111 (1.5%) as the most prevalent non-O157 STEC serovars in retail food and cattle samples in Egypt. Our initial work in this area [18] identified the incidence of O26:H11 and O121:H7 in heat-treated dairy products. The endemic persistence of identical *E. coli* lineages across various ready-to-consume fermented milk products marketed within the same geographical area indicates a lack of hygiene control measures at the farm, processing, or retail levels, facilitating the food-chain and cross-border circulation of specific strains. Thus, Dehkordi et al. [37] reported that O26 prevalence reached 12% among the fermented dairy products examined in Iran, with yogurt identified as the most contaminated dairy product. Furthermore, other serotypes (O153:H2, O125:H21, O119:H6, O103:H2, O127:H6, and O55:H7) were less frequently identified.

Indeed, molecular characterization of key virulence determinants (e.g., *stx*, *eae*, *hlyA*) that contribute to *E. coli* survival and pathogenicity is critical for documenting that isolated serogroups harbor high-risk pathotypes [40,41,42]. The Shiga toxin (*stx*) gene mediates the knockdown of host protein synthesis, leading to microvillus degeneration, atrophy, and complications of hemorrhagic colitis and HUS [43]. The intimin (*eae*) gene mediates firm bacterial adhesion to enterocytes and the injury of microvilli [44]. The α-hemolysin (*hlyA*) gene is a pore-forming toxin that contributes to erythrocyte and host cell lysis [18,19,45]. In the current study, the prevalence of *E. coli*-harboring *stx1* and *stx2* was significantly higher (76.5%) when compared with a previous surveillance by Ombarak et al. [27], who reported that *stx1* and *stx2* were detected in only 0.9% and 0.45% of raw milk marketed in the Nile Delta region of Egypt, specifically the Menofia and El Beheira Governorates. Elbastawesy et al. [31] identified adhesion genes (*lpfAO113* and *ehaA*) as prevalent in dairy products marketed across the Nile Delta area, whereas toxin genes were uncommon. A surge in the incidence of STEC strains in investigated yogurt and rayeb milk represents a burden on healthcare systems and indicates deteriorating sanitation practices and widespread dissemination of virulent clones within the dairy-processing chain.

The current view is that the presence of *eaeA* and plasmid-encoded *hlyA* has been identified as a hallmark of enterohemorrhagic *E. coli* (EHEC), enterotoxigenic (ETEC), shigatoxigenic (STEC), and enteropathogenic *E. coli* (EPEC) [19,46]. Our study reported limited isolates carrying *eaeA* (17.6%) and *hlyA* (32.4%), with most *stx*-positive strains lacking *eaeA*. In contrast, Elzhraa et al. [18] found that *eaeA* was ubiquitous in *E. coli* isolated from dairy products in Mansoura. Nonetheless, isolates harboring *stx1* or *stx2* received particular attention because Shiga toxin genes alone are independently sufficient to induce severe clinical outcomes [40]. A notable exception is that six isolates (17.6%) belonged to the classic EHEC genotypes and carried the full virulence set (*stx1*, *stx2*, *eaeA*, and *hlyA*), which is consistent with global reviews that emphasize the importance of these genes in *E. coli* pathogenicity [41,42]. One study reported that *E. coli* from milk and/or dairy products frequently carries *eaeA*, *stx1/2*, and *hlyA*, a finding that is broadly consistent with our profile of predominantly Shiga-toxigenic strains [45]. This is a highly interesting but so far unexplored aspect of clinical relevance.

From a public health perspective, the AMR traits augment the pathogenic potential of food-borne pathogens and further complicate clinical management and treatment outcomes of systemic infections in humans [47,48]. The isolates exhibited extensive AMR (80–85%) to penicillins, tetracycline, and chloramphenicol, as well as high resistance to aminoglycosides (kanamycin). The elevated levels of resistance to β-lactams and tetracyclines observed in the current study are consistent with findings from other STEC surveys. For instance, Ranjbar et al. [45] found that more than 95% of STEC isolates recovered from dairy products in Iran were resistant to ampicillin and tetracycline. Elmonir et al. [26] reported that approximately 48.6% of Egyptian STEC isolates exhibited resistance to ampicillin, whereas resistance to aminoglycosides, including gentamicin, was threefold lower than our findings in *E. coli* isolated from cattle samples. Despite this, *E. coli* isolates in the current study demonstrated high susceptibility to aminoglycosides (neomycin and streptomycin), macrolides (azithromycin), and aztreonam. Elmonir et al. [26] found that approximately 51% of STEC were MDR. Currently, the high percentage of resistance to clinically available antibiotics, with 73.5% of isolates being MDR and 26.5% being XDR, highlights the emergence of MDR strains, which is worrisome and reflects the assertion that raw milk and yogurt can harbor highly resistant pathogens, compromising the effectiveness of standard therapeutic interventions in clinical settings. Of note, O153:H2 (52) obtained from rayeb milk was labeled XDR due to its susceptibility to at least one antimicrobial class and reached an MAR index of 0.813, indicating that this isolate is of particular concern and reflects contamination of the examined dairy products. The MAR indices > 0.2 for most isolates indicate high-risk contamination sources in the dairy supply system, potentially hazardous to humans [49]. Krumperman [50] suggested that *E. coli* isolates with MAR values ≥0.2 may reflect fecal contamination from humans, livestock, or associated environments, likely driven by plasmid-mediated resistance exchange between *E. coli* and other coliform bacteria in wastewater systems. However, this cutoff is empirical and somewhat arbitrary; values around 0.20–0.25 can represent heterogeneous contamination scenarios and should be interpreted cautiously. In food matrices, elevated MAR indices warrant further investigation, even when bacterial counts remain within acceptable limits. Evidence is emerging that MDR-STEC in everyday fermented dairy products poses a real public health risk [51]. The present study further extends this concern by demonstrating that yogurt and rayeb milk in Dakahlia can serve as reservoirs of the EHEC serogroup of *E. coli*. Subsequently, implementing rigorous manufacturing and handling practices, such as the Hazard Analysis and Critical Control Points (HACCP) system, is imperative to ensure product safety and integrity. Furthermore, there is an urgent need to improve hygiene and conduct hazard analysis at the Mansoura dairy-processing facility.

The phenotypic AMR profile indicates that the bacteria survive antibiotics, but it does not clarify the specific genetic mechanisms responsible for this resistance. Presumably, knowing AMR genes helps detect regional or global spread of resistant strains and supports food safety surveillance strategies [52,53,54]. All 34 *E. coli* isolates carried at least one of the *kan*, *dfrA*, *blaOXA-1*, *tetA(A)*, and *vanA* AMR genes, thereby emphasizing the pervasive nature of resistant strains in the food chain. A striking similarity is observed between this study and that of Ombarak et al. [27], who conducted a survey of Egyptian raw milk and cheese and reported that *kan*, *dfrA*, *blaOXA-1*, and *tetA* were identified as the predominant kanamycin, trimethoprim, β-lactamase, and tetracycline resistant genes in 73.5% (163/222), 67.6% (150/222), 61.8% (137/222), and 23.87% (53/222) of *E. coli* isolates, respectively. An interesting open issue is that research conducted in Egypt has repeatedly highlighted the widespread dissemination of β-lactamase-encoding genes among dairy-derived *E. coli*. Ombarak et al. [27] reported the frequent detection of *blaTEM* and *blaCTX-M* genes in ampicillin-resistant isolates from milk. In a broader context, Braun et al. [55] conducted a surveillance study in Nile Delta dairy farms and confirmed the prevalence of ESBL determinants, where 113 out of 114 cephalosporin-resistant *E. coli* isolates were found to harbor at least one ESBL gene, predominantly *bla_CTX-M* (*n* = 105), followed by *bla_TEM* (*n* = 90) and *bla_SHV* (*n* = 1). In a similar vein, Ibrahim et al. [56] identified *blaCTX-M* (*n* = 12/53), *blaTEM* (*n* = 4/53), and *blaOXA* (*n* = 2/53) among dairy farm isolates, thereby demonstrating the genetic diversity of β-lactamases circulating in Egyptian dairy systems. These findings declare early evidence of *E. coli*-harboring ESBLs disseminating in ready-to-consume dairy products. Recently, Ashraf et al. [57] identified *blaTEM* in all MDR *E. coli* strains recovered from milk, with additional carriage of *blaCTX-M* in selected isolates. This report further emphasizes the expansion of ESBL genotypes within the dairy chain. Hence, the high prevalence of *blaOXA-1* among yogurt and rayeb milk isolates in the current study is consistent with earlier findings confirming that β-lactam resistance is deeply entrenched in Egypt’s dairy production environments. Of note, the frequent co-occurrence of *dfrA* with aminoglycoside genes (as noted in the present study) is echoed by their report of class-1 integrons carrying *dfrA12* and *aadA* cassettes [58], indicating the presence of mobile elements. Although we did not specifically assay integrons, the results strongly suggest horizontal gene transfer.

Evidence has accumulated that *E. coli* strains are naturally resistant to vancomycin due to the impermeability of the outer membrane [59]. It is noteworthy that almost half of the *E. coli* isolates examined in this study carried the *vanA* gene. This finding is of particular interest, as the detection of *vanA*-positive isolates suggests the horizontal acquisition of glycopeptide resistance determinants from other bacteria [60] or plasmid exchange between *E. coli* and other bacteria in high-risk environments such as sewage or wastewater [50]. Despite this intrinsic resistance to vancomycin and positivity for the *vanA* AMR gene, 52.9% of isolates were susceptible to the VAN antibiotic disc, suggesting that harboring the gene does not ultimately guarantee expression or functional activity in mediating in vitro microbial killing [61]. A defect in intrinsic resistance is associated with alterations in cell wall metabolism and remodeling enzymes, such as lytic transglycosylases (Slt and MltG), which influence interactions with penicillin-binding proteins (PBPs) and may modify intrinsic antibiotic resistance [62]. Such VAN resistance traits in our dataset are consistent with Abd Al-Kareem et al. [63], who found that among 15 vancomycin-resistant *E. coli* isolates from various water sources in Baghdad, eight carried the *vanA* gene and one carried the *vanB* gene. Furthermore, Sagban and Jawad Al-Zubaidi [64] discovered that the occurrence rates of both *vanA* and *vanB* genes were 36% and 16%, respectively, in *E. coli* samples isolated from clinical and water sources in Baqubah city. Comparable findings have been reported from other regions, extending beyond Egypt’s geographical confines. As documented by Timofeeva et al. [65], *vanA*-positive *E. coli* was observed at varying frequencies across Russian regions, with the Vologda region recording 8.2%, the Udmurt Republic 63.2%, and the Krasnoyarsk region 34.8% prevalence. The same study also identified *vanB* in 6.6%, 31.6%, and 13.9% of isolates in these regions, respectively. The co-detection of both genes within the same isolate was rare, occurring in only 2.6% and 0.9% of cases in Vologda and Krasnoyarsk, respectively. These observations, in conjunction with our finding of an unusually high prevalence of *vanA* in dairy-derived *E. coli* from Dakahlia, not only suggest that the gene may be more mobile than previously assumed but also indicate possible spread through environmental reservoirs and horizontal gene transfer events in livestock-associated settings.

## 4. Materials and Methods

### 4.1. Study Design and Sample Processing

Over two Ramadan seasons (March–April 2023, and March–April 2025), a total of 150 freshly produced fermented dairy samples, comprising 75 Rayeb milk and 75 yogurt samples (30 samples per city: 15 Rayeb and 15 yogurt), were randomly collected from supermarkets and retailers located in highly populated cities (Mansoura, El Senbellawein, Dekernes, Belqas, and Mit Ghamr) of Dakahlia governorate, Egypt (https://www.citypopulation.de/en/egypt/admin/ (accessed on 20 March 2026)), and were transported to the laboratory under refrigeration within 2 h. From this initial screening, 34 *E. coli* isolates were successfully recovered (11 from rayeb milk and 23 from yogurt) and subjected to further analysis. The sample size was statistically calculated and determined a priori based on the objectives of prevalence estimation and comparative analysis, which commonly employed sample sizes ranging from 120 [66,67,68] to 125 [28] for investigating *E. coli* prevalence and characterization in fermented dairy products.

Samples were prepared for *E. coli* isolation according to the method of Yang and Yoon [69]. In brief, about 25 g of each sample was homogenized in 225 mL of nutrient broth (Cat. No.: 1.05443.0500, Merck KGaA, Darmstadt, Germany) using a Stomacher blender (Seward, Worthing, UK). Then, 1 mL of the homogenate was ten-fold serially diluted with 0.1% peptone water, enriched in MacConkey broth, and incubated at 35 ± 2 °C for 24 ± 2 h. Subculturing was performed by plating on MacConkey agar (Cat. No.: MCE20500, Biolab Inc., Budapest, Hungary), and the plates were examined after incubation at 37 °C for 24 h [70]. Presumptive colonies were preliminarily identified as *E. coli* through 20 biochemical tests provided within API 20E strips (bioMérieux, Marcy-l’Étoile, France). Rapid and reliable species-level identification of isolates was assessed using MALDI-TOF MS (Bruker Daltonics, Bremen, Germany). Based on the mass spectral profiles of *E. coli* intracellular proteins, a MALDI-TOF MS score ≥ 2.0 was considered confirmatory [71]. Serotyping was performed according to the manufacturer’s updated protocol and the method described by Beutin et al. [72], using O and H antisera from Denka Seiken (Tokyo, Japan). Isolates were preserved in Tryptic Soy Broth (TSB, Cat. No.: TSB20500, Biolab Inc., Budapest, Hungary) with 20% glycerol at –80 °C [37], for the retrospective phenotyping of antimicrobial susceptibility and molecular characterization.

### 4.2. Phenotypic Characterization

Antimicrobial susceptibility was performed using the disk diffusion method of Bauer et al. [73]. Cultures of *E. coli* were incubated overnight in TSB (Cat. No.: TSB20500, Biolab Inc., Budapest, Hungary) at 37 °C, and the density of bacterial suspensions in PBS (phosphate-buffered saline, pH 7.4) was adjusted to 0.5 McFarland turbidity using a densitometer (Den-1, Biosan, Riga, Latvia). The suspension was evenly spread onto Mueller–Hinton Agar (MHA, Cat No.: MHT20500, Biolab Inc., Budapest, Hungary), and 16 commercially available Bio-Rad^®^ antibiotic discs (Bio-Rad, Marnes-la-Coquette, France; Table 2) were placed at appropriate distances to prevent overlapping inhibition zones. The selection of these antibiotics was based on their frequent use in research and clinical cases in both veterinary and human medicine [74,75]. Plates were incubated at 37 °C for 24 h, and the results were interpreted according to the approved Clinical and Laboratory Standards Institute (CLSI) disc breakpoint datasets [sensitive (S), resistant (R), or intermediate (I)] where applicable [76]. Antibiotics lacking CLSI interpretive criteria for *E. coli* (PEN, OXC, VAN, NEO, and ERY) were included for comparative and epidemiological purposes based on their reported use in veterinary studies related to food of animal origin [74,75] and clinical human studies (considering inhibition zone diameters and clinical outcomes in human patients) [77,78,79]. As standardized breakpoints have not been established for these antibiotics in *E. coli*, the corresponding results should be interpreted with caution and may not reflect clinical efficacy [80]. The Multiple Antibiotic Resistance (MAR) index was determined as a/b, where “a” is the number of antibiotics the isolate was resistant to and “b” is the total number of antibiotics tested [50].

### 4.3. Determination of the Presence of Virulence and Antimicrobial Resistance Genes

Based on silica membrane spin-column technology, genomic DNA (gDNA) was extracted from the retrieved isolates using the QIAamp^®^ DNA Mini Kit (Cat No.: 51304, Qiagen, Hilden, Germany) according to the manufacturer’s instructions. In brief, the protease enzyme was added to the prepared sample at a 1:10 (*v*/*v*) ratio, vortexed in AL buffer, and incubated at 56 °C for 10 min to enhance cell lysis without gDNA degradation. About 200 μL of absolute ethanol was added to the lysate mixture after brief centrifugation to maximize gDNA precipitation and binding to the silica membrane. For maximum gDNA binding, the lysate mixture was added to a QIAamp spin column, centrifuged at 3500× *g* for 1 min, and washed with buffers AW1 and AW2 to preserve DNA binding and remove impurities. In a clean microcentrifuge tube, 100 μL of buffer AE was added to release gDNA from silica interactions. The purity and suitability of the eluted DNA for molecular analyses were assessed using UV spectrophotometry (NanoDrop^®^ ND-1000, Nanodrop Technologies, Wilmington, DE, USA) by determining the A260/A280 (DNA/protein contamination) and A260/A230 (DNA/organic compound and salt contamination) ratios. Integrity was visualized by agarose gel electrophoresis.

In accordance with Raimondi et al. [81], PCR amplification of selected virulence and AMR genes was assayed using EmeraldAmp^®^ GT PCR Master Mix (Cat No.: RR310A, Takara, Paris, France). The total reaction volume was 25 μL, containing 2× master mix (12.5 μL), template DNA (5 μL) at 50 ng/µL, 1 μL each of forward and reverse primers (20 pmol, Table 3), and 5.5 μL PCR-grade water. Amplification cycling conditions in a DNA thermocycler (Eppendorf Mastercycler, Eppendorf-Nethel-Hinz GmbH, Hamburg, Germany) were 5 min of initial denaturation at 94 °C, 35 cycles of denaturation for 30 s at 94 °C, annealing at 58 °C for 40 s (for multiplex virulence gene amplification) or gene-specific annealing temperatures (Table 3) for uniplex reactions, and extension at 72 °C for 45 s, with a final extension at 72 °C for 10 min. According to Sheth [82], M-PCR was performed for the simultaneous detection of virulence genes (*eaeA*, *stx1*, *stx2*, and *hlyA*) in a single reaction, while AMR genes (*kan*, *dfrA*, *blaOXA-1*, *tetA(A)*, and *vanA*) were amplified individually using U-PCR. To avoid primer competition, the annealing temperature for M-PCR was selected based on all primer pairs’ melting temperatures and preliminary optimization trial runs using combined primer sets across a range of temperatures, with 58 °C yielding consistent amplification of all target genes and clear bands at the expected amplicon sizes (890, 779, 614, and 165 bp) without additional non-specific bands amplification. All PCR products were separated by electrophoresis on 1.5% (*w*/*v*) agarose gel in 1× TAE buffer (pH 8.3) containing 0.5 μg/mL ethidium bromide, run at 1–5 V/cm for 30 min alongside 6 μL of a 100 bp DNA ladder (Cat No.: SM0243, Fermentas, Thermo Scientific, Waltham, MA, USA). Bands’ specificity and expected sizes were visualized under UV light and documented by photography. Isolates harboring the target gene were used as positive controls to validate PCR amplification conditions, ensure primer specificity, and maintain reproducibility of the molecular detection protocol. In line with Radi et al. [83], well-characterized *E. coli* O157:H7 (ATCC 43895, supplied by AHRI of Egypt, Giza, Egypt) was used as a positive control for virulence gene amplification (*stx1*, *stx2*, *eaeA*, and *hlyA*). For targeted AMR genes, previously PCR-validated *E. coli* isolates in our lab were used as positive controls for *blaOXA-1*, *tetA(A)*, *dfrA*, and *kan*. As per Maharjan et al. [84], *Enterococcus faecalis* (ATCC 51299, supplied by AHRI of Egypt) was used as a positive reference strain for the *vanA* gene during gel electrophoresis. The negative control used a template-free reaction to exclude contamination.

### 4.4. Data Analysis and Illustration

The sample size was primarily determined based on previous studies in fermented dairy products conducted in similar settings in Egypt and nearby regions. Assuming an expected *E. coli* prevalence of 20% (*p* = 0.20), a 95% confidence level (Z = 1.96), and a margin of error of ±10% (E = 0.10), the minimum required sample size per group was calculated using the single-proportion formula:

$$n=\frac{Z^{2}\times p(1-p)}{E^{2}}=\frac{\left(1.96{)}^{2}\times 0.20\times 0.80\right.}{{\left(0.10\right)}^{2}}\approx 61$$

The calculated sample size (*n* = 61) was increased to 75 per group (total *n* = 150) to improve precision, allow for comparison between yogurt and rayeb milk, and account for potential sample loss. A post hoc power analysis further confirmed that this study achieved approximately 83% power to detect this difference at α = 0.05, supporting the adequacy of the selected sample size. Additionally, power analysis was evaluated using G*Power software (v3.1.9.4), assuming a large effect size (Cohen’s d = 0.80), α = 0.05, and power (1 − β) = 0.90, confirming that the selected sample size is adequate for detecting meaningful differences between sampled products.

Percentage differences in the distribution of serotypes across the investigated products were determined using Fisher’s exact test (for serovars with zero counts in either of two products) and the chi-squared (χ^2^) test in SPSS Version 20 (Chicago, IL, USA). Statistical significance was set at *p* < 0.05. The distribution percentage of isolates across surveyed regions and collected samples was visualized as a Doughnut chart in OriginPro (version 2022, OriginLab, Northampton, MA, USA). The phenotypic AMR profile and patterns of virulence and AMR genes across identified serotypes were illustrated as a complex heatmap with hierarchical clustering using the “Complex Heatmap, version 2.15.4” and “pheatmap, version 1.0.12” packages in RStudio version 4.3.1 (https://www.r-project.org/). Clustering was performed using Euclidean distance as the dissimilarity measure and Ward’s linkage method to group samples and variables. Susceptibility (R, I, and S) percentages to selected antibiotic discs were illustrated as stacked bar plots in RStudio using the “ggplot2, version 4.0.1” and “ggthemes, version 5.0.0” packages. Chord plots were illustrated in OriginPro 2022 (OriginLab).

## 5. Conclusions

The present study further supports the use of *E. coli* as a relevant indicator organism for global monitoring of AMR and the dissemination of virulence- and resistance-associated genes within the dairy food chain. The findings are particularly pertinent for fermented dairy products marketed in Dakahlia Governorate (Egypt), especially during periods of increased consumption and production (e.g., Ramadan), when strict hygiene control measures may be more difficult to implement. This work has identified rayeb milk and yogurt as the primary reservoirs of virulent, MDR, and XDR *E. coli* strains in densely populated areas, contributing to a One Health perspective. Notably, virulence genes *stx1* and *stx2* were detected in 76.47% of isolates, while *eaeA* and *hlyA* were identified in O26:H11, O119:H6, and O55:H7 serotypes, indicating the presence of potentially pathogenic strains. A high burden of AMR was observed, with 73.53% (25/34) of isolates classified as MDR and 26.47% (9/34) as XDR. At the molecular level, the widespread occurrence of *E. coli* resistant to clinically available antimicrobials and positive to AMR determinants [*tetA(A)* (100%), *kan* (70.59%), *dfrA* (67.65%), *blaOXA-1* (61.76%), and *vanA* (47.06%)] was detected in isolates from fermented milk products. These findings further suggest the urgent need for enhanced surveillance and stricter hygiene measures, particularly during periods of intensified seasonal production of fermented foods. They also underscore the public health risk posed by contaminated ready-to-consume fermented milk products as a critical interface between animal-derived foods and human consumers within a One Health framework. Improved insights into these aspects will ultimately be necessary for farmer education, improved cold-chain handling, and robust antimicrobial stewardship in Egypt’s Delta region, where smallholder farms and informal markets dominate the dairy supply chain. This study has some limitations. The detection of resistance genes, particularly *vanA*, was based on PCR without sequencing confirmation, and should therefore be interpreted cautiously. The absence of a non-Ramadan comparison group precludes definitive attribution of findings to seasonal effects. Additionally, the relatively small sample size per city and product (*n* = 15) may limit the generalizability of location-specific trends, despite an adequate overall sample size (*n* = 150).

## Figures and Tables

**Table 1:** Distribution of *E. coli* serovars in rayeb and yogurt samples.

Serovar (O:H)	Isolates (*n* = 34)	Rayeb (*n* = 11)	Yogurt (*n* = 23)	χ^2^	*p*-Value
O153:H2	7 (20.59%)	3 (27.3%)	4 (17.4%)	0.05	0.831
O125:H21	5 (14.7%)	0 (0.0%)	5 (21.7%)	1.34	0.247
O119:H6	5 (14.7%)	1 (9.1%)	4 (17.4%)	0.01	0.903
O111:H2	4 (11.7%)	0 (0.0%)	4 (17.4%)	0.82	0.366
O26:H11	4 (11.7%)	4 (36.4%) *	0 (0.0%)	6.30	0.012
O127:H6	4 (11.7%)	3 (27.3%)	1 (4.3%)	1.88	0.170
O103:H2	3 (8.8%)	0 (0.0%)	3 (13.0%)	0.37	0.543
O55:H7	2 (5.9%)	0 (0.0%)	2 (8.7%)	0.05	0.819

* Significant difference at *p* < 0.05.

**Table 2:** List of applied antibiotics for the phenotypic antimicrobial sensitivity test.

Classification	Antibiotics	Potency (μg/disc)	Classification	Antibiotics	Potency (µg/disc)
Penicillins	PEN	6	Aminoglycosides	HLG	120
	AMP	10		HLS	300
	OXC	1		NEO	30
DHFR inhibitors	TMP	5		KMN	30
Amphenicols	CHL	30	Lincosamides	CMN	2
			Tetracyclins	TET	30
Monobactams	ATM	30	Fluorquinolones	CP	5
Glycopeptides	VAN	30	Macrolides	ERY	15
				AZM	15

PEN, Penicillin; AMP, Ampicillin; OXC, Oxacillin; TMP, Trimethoprim; CHL, Chloramphenicol; ATM, Aztreonam; VAN, Vancomycin; HLG, High-Load Gentamicin; HLS, High-Load Streptomycin; NEO, Neomycin; KMN, Kanamycin; CMN, Clindamycin; TET, Tetracycline; CP, Ciprofloxacin; ERY, Erythromycin; AZM, Azithromycin.

**Table 3:** The primer pairs used for the amplification of targeted virulence and AMR determinants, along with the annealing temperature and amplicon size for each primer set.

Gene Groups	Target Genes	Direction	Nucleotide Sequence (5′ > 3′)	Annealing Temp.	Amplicon Size (bp)	Reference
Virulence	*stx1*	F	ACACTGGATGATCTCAGTGG	58 °C	614	[85]
		R	CTGAATCCCCCTCCATTATG			
	*stx2*	F	CCATGACAACGGACAGCAGTT	58 °C	779	
		R	CCTGTCAACTGAGCAGCACTTTG			
	*eaeA*	F	GTGGCGAATACTGGCGAGACT	58 °C	890	[86]
		R	CCCCATTCTTTTTCACCGTCG			
	*hlyA*	F	ACGATGTGGTTTATTCTGGA	58 °C	165	[20]
		R	CTTCACGTGACCATACATAT			
AMR	*kan*	F	GTGTTTATGGCTCTCTTGGTC	54 °C	621	[87]
		R	CCGTGTCGTTCTGTCCACTCC			
	*dfrA*	F	TGGTAGCTATATCGAAGAATGGAGT	60 °C	425	[88]
		R	TATGTTAGAGGCGAAGTCTTGGGTA			
	*tetA(A)*	F	GGTTCACTCGAACGACGTCA	50 °C	570	[89]
		R	CTGTCCGACAAGTTGCATGA			
	*blaOXA-1*	F	TCAACTTTCAAGATCGCA	54 °C	609	[90]
		R	GTGTGTTTAGAATG GTGA			
	*vanA*	F	GGCAAGTCAGGTGAAGATG	54 °C	763	[84]
		R	ATCAAGCGGTCAATCAGTTC			

*stx1*, Shiga toxin 1 gene; *stx2*, Shiga toxin 2 gene; *eaeA*, intimin (attaching and effacing) gene; *hlyA*, hemolysin A gene; *kan*, kanamycin resistance gene; *dfrA*, dihydrofolate reductase gene (trimethoprim resistance); *tetA(A)*, tetracycline resistance gene; *blaOXA-1*, OXA-1 β-lactamase gene; *vanA*, vancomycin resistance gene; F, forward primer; R, reverse primer.

## Data Availability

The original contributions presented in this study are included in this article (and Supplementary Materials); further inquiries can be directed to the corresponding author.

## Supplementary Materials

The following supporting information can be downloaded at: https://www.mdpi.com/article/10.3390/antibiotics15050483/s1, Table S1: Antimicrobial resistance profiles, serotypes, and multiple antibiotic resistance (MAR) indices of *E.coli* isolates (*n* = 34) recovered from fermented dairy products (rayeb milk and yogurt). File S1: MALDI-TOF data; File S2: Raw data; File S3: Regional distribution; File S4: Gel images of virulence and AMR genes visualization.
