# Supplementary material for: Virulence and Antimicrobial Resistance Traits of Escherichia coli Retrieved from Fermented Dairy Products During Ramadan in Egypt: Seasonal Public Health Implications
Source: Antibiotics (Basel). 2026 May 9;15(5):483. doi: 10.3390/antibiotics15050483 (PMC13203909; doi:10.3390/antibiotics15050483)
Supplement: Supplementary file 1 [file antibiotics-15-00483-s001.zip › File S1-Maldi Toff.pdf]

Bruker MALDI Biotyper Identification Results

HUNGARIAN UNIVERSITY OF AGRICULTURE AND LIFE SCIENCES

Run Info:

Run Identifier: 250226-1228-101
Comment:
Operator: Admin@MALDITOF
Run Creation Date/Time: 2025-02-26T12:42:05.524
Number of Tests: 18
Type: Standard
BTS-QC: not present
BTS-QC Position:
Instrument ID: 8269944.03839
Server Version: 4.1.100 (PYTH) 174 2019-06-158_01-16-09

Result Overview

Sample Name	Sample ID	Organism (best match)	Score Value	Organism (second-best match)	Score Value
E12 (+++) (A)	1 (Standard)	Escherichia coli	2.26	Escherichia coli	2.23
F1 (+++) (A)	2 (Standard)	Escherichia coli	2.23	Escherichia coli	2.22
F2 (+++) (A)	3 (Standard)	Escherichia coli	2.30	Escherichia coli	2.28
F3 (+++) (A)	7 (Standard)	Escherichia coli	2.44	Escherichia coli	2.41
F4 (+++) (A)	8 (Standard)	Escherichia coli	2.46	Escherichia coli	2.45
F5 (+++) (A)	16 (Standard)	Bacillus thuringiensis	2.03	Bacillus mycoides	1.95
F6 (+++) (A)	25 (Standard)	Escherichia coli	2.52	Escherichia coli	2.35

Result overview table--continued on next page

Sample Name	Sample ID	Organism (best match)	Score Value	Organism (second-best match)	Score Value
Result overview table--continued from previous page					
F7 (+++) (A)	26 (Standard)	Escherichia coli	2.51	Escherichia coli	2.38
F8 (+++) (A)	29 (Standard)	Escherichia coli	2.44	Escherichia coli	2.40
F9 (+++) (A)	47 (Standard)	Escherichia coli	2.36	Escherichia coli	2.25
F10 (+++) (A)	49 (Standard)	Escherichia coli	2.43	Escherichia coli	2.36
F11 (+++) (C)	52 (Standard)	Escherichia coli	2.36	Escherichia coli	2.33
F12 (+++) (C)	52 (Standard)	Escherichia coli	2.24	Escherichia coli	2.07
G1 (+++) (A)	57 (Standard)	Escherichia coli	2.44	Escherichia coli	2.44
G2 (+++) (A)	58 (Standard)	Escherichia coli	2.36	Escherichia coli	2.31
G3 (+++) (A)	62 (Standard)	Escherichia coli	2.20	Escherichia coli	2.18
G4 (+++) (A)	64 (Standard)	Escherichia coli	2.36	Escherichia coli	2.28
G5 (+++) (A)	64 (Standard)	Escherichia coli	2.49	Escherichia coli	2.39

Compressed View

Sample 1

Sample Name: E12
Sample Description:
Sample ID: 1
Sample Creation Date/Time: 2025-02-26T12:30:42.427
Sample Type: Standard
Identification Method: MALDI Biotyper MSP Identification Standard Method 1.1
Preprocessing Method: MALDI Biotyper Preprocessing Standard Method 1.1
ACQ Method: D:\Methods\flexControlMethods\MBT_FC.par
ACQ Timestamp: 2025-02-26T12:43:16.981
AutoXecute Method: MBT_AutoX
Applied Taxonomy Tree: Taxonomy, Bruker Taxonomy, Projects, Sajat izolatumok

Escherichia coli	10 hits	2.26	2.23	2.20	2.20	2.19	2.12	2.05	2.03	2.01	2.00

Sample 2

Sample Name: F1
Sample Description:
Sample ID: 2
Sample Creation Date/Time: 2025-02-26T12:30:42.429
Sample Type: Standard
Identification Method: MALDI Biotyper MSP Identification Standard Method 1.1
Preprocessing Method: MALDI Biotyper Preprocessing Standard Method 1.1
ACQ Method: D:\Methods\flexControlMethods\MBT_FC.par
ACQ Timestamp: 2025-02-26T12:32:53.971
AutoXecute Method: MBT_AutoX
Applied Taxonomy Tree: Bruker Taxonomy, Taxonomy, Sajat izolatumok, Projects

Escherichia coli	10 hits	2.23	2.22	2.19	2.16	2.16	2.07	2.01	1.99	1.97	1.95

Sample 3

Sample Name: F2
Sample Description:
Sample ID: 3
Sample Creation Date/Time: 2025-02-26T12:30:42.431
Sample Type: Standard
Identification Method: MALDI Biotyper MSP Identification Standard Method 1.1
Preprocessing Method: MALDI Biotyper Preprocessing Standard Method 1.1
ACQ Method: D:\Methods\flexControlMethods\MBT_FC.par
ACQ Timestamp: 2025-02-26T12:33:18.451
AutoXecute Method: MBT_AutoX
Applied Taxonomy Tree: Sajat izolatumok, Projects, Bruker Taxonomy, Taxonomy

Escherichia coli	10 hits	2.30	2.28	2.26	2.22	2.20	2.20	2.06	2.05	2.03	1.96

Sample 4

Sample Name: F3
Sample Description:
Sample ID: 7
Sample Creation Date/Time: 2025-02-26T12:30:42.434
Sample Type: Standard
Identification Method: MALDI Biotyper MSP Identification Standard Method 1.1
Preprocessing Method: MALDI Biotyper Preprocessing Standard Method 1.1
ACQ Method: D:\Methods\flexControlMethods\MBT_FC.par
ACQ Timestamp: 2025-02-26T12:33:49.467
AutoXecute Method: MBT_AutoX
Applied Taxonomy Tree: Sajat izolatumok, Projects, Bruker Taxonomy, Taxonomy

Escherichia coli	10 hits	2.44	2.41	2.31	2.28	2.28	2.18	2.18	2.17	2.16	2.13

Sample 5

Sample Name: F4
Sample Description:
Sample ID: 8
Sample Creation Date/Time: 2025-02-26T12:30:42.436
Sample Type: Standard
Identification Method: MALDI Biotyper MSP Identification Standard Method 1.1
Preprocessing Method: MALDI Biotyper Preprocessing Standard Method 1.1
ACQ Method: D:\Methods\flexControlMethods\MBT_FC.par
ACQ Timestamp: 2025-02-26T12:34:14.115
AutoXecute Method: MBT_AutoX
Applied Taxonomy Tree: Taxonomy, Bruker Taxonomy, Projects, Sajat izolatumok

Escherichia coli	10 hits	2.46	2.45	2.41	2.33	2.29	2.23	2.20	2.13	2.11	2.10

Sample 6

Sample Name: F5
Sample Description:
Sample ID: 16
Sample Creation Date/Time: 2025-02-26T12:30:42.438
Sample Type: Standard
Identification Method: MALDI Biotyper MSP Identification Standard Method 1.1
Preprocessing Method: MALDI Biotyper Preprocessing Standard Method 1.1
ACQ Method: D:\Methods\flexControlMethods\MBT_FC.par
ACQ Timestamp: 2025-02-26T12:34:47.154
AutoXecute Method: MBT_AutoX
Applied Taxonomy Tree: Projects, Sajat izolatumok, Taxonomy, Bruker Taxonomy

Bacillus thuringiensis	1 hit	2.03									
Bacillus mycoides	3 hits		1.95	1.92		1.88					
Bacillus cereus	2 hits			1.92			1.87				
Bacillus pseudomycoides	1 hit							1.65			
Acinetobacter towneri	1 hit								1.55		
Bacillus cytotoxicus	1 hit									1.51	
Neisseria meningitidis	1 hit										1.50

Sample 7

Sample Name: F6
Sample Description:
Sample ID: 25
Sample Creation Date/Time: 2025-02-26T12:30:42.441
Sample Type: Standard
Identification Method: MALDI Biotyper MSP Identification Standard Method 1.1
Preprocessing Method: MALDI Biotyper Preprocessing Standard Method 1.1
ACQ Method: D:\Methods\flexControlMethods\MBT_FC.par
ACQ Timestamp: 2025-02-26T12:35:13.934
AutoXecute Method: MBT_AutoX
Applied Taxonomy Tree: Projects, Bruker Taxonomy, Sajat izolatumok, Taxonomy

Escherichia coli	10 hits	2.52	2.35	2.23	2.22	2.21	2.16	2.12	2.09	2.08	2.04

Sample 8

Sample Name: F7
Sample Description:
Sample ID: 26
Sample Creation Date/Time: 2025-02-26T12:30:42.443
Sample Type: Standard
Identification Method: MALDI Biotyper MSP Identification Standard Method 1.1
Preprocessing Method: MALDI Biotyper Preprocessing Standard Method 1.1
ACQ Method: D:\Methods\flexControlMethods\MBT_FC.par
ACQ Timestamp: 2025-02-26T12:35:31.715
AutoXecute Method: MBT_AutoX
Applied Taxonomy Tree: Sajat izolatumok, Projects, Bruker Taxonomy, Taxonomy

Escherichia coli	10 hits	2.51	2.38	2.34	2.27	2.26	2.25	2.22	2.20	2.19	2.16

Sample 9

Sample Name: F8
Sample Description:
Sample ID: 29
Sample Creation Date/Time: 2025-02-26T12:30:42.446
Sample Type: Standard
Identification Method: MALDI Biotyper MSP Identification Standard Method 1.1
Preprocessing Method: MALDI Biotyper Preprocessing Standard Method 1.1
ACQ Method: D:\Methods\flexControlMethods\MBT_FC.par
ACQ Timestamp: 2025-02-26T12:36:08.292
AutoXecute Method: MBT_AutoX
Applied Taxonomy Tree: Sajat izolatumok, Bruker Taxonomy, Projects, Taxonomy

Escherichia coli	10 hits	2.44	2.40	2.36	2.30	2.26	2.26	2.17	2.16	2.14	2.12

Sample 10

Sample Name: F9
Sample Description:
Sample ID: 47
Sample Creation Date/Time: 2025-02-26T12:30:42.448
Sample Type: Standard
Identification Method: MALDI Biotyper MSP Identification Standard Method 1.1
Preprocessing Method: MALDI Biotyper Preprocessing Standard Method 1.1
ACQ Method: D:\Methods\flexControlMethods\MBT_FC.par
ACQ Timestamp: 2025-02-26T12:37:13.396
AutoXecute Method: MBT_AutoX
Applied Taxonomy Tree: Sajat izolatumok, Projects, Bruker Taxonomy, Taxonomy

Escherichia coli	10 hits	2.36	2.25	2.24	2.23	2.23	2.13	2.10	2.09	2.08	2.08

Sample 11

Sample Name: F10
Sample Description:
Sample ID: 49
Sample Creation Date/Time: 2025-02-26T12:30:42.451
Sample Type: Standard
Identification Method: MALDI Biotyper MSP Identification Standard Method 1.1
Preprocessing Method: MALDI Biotyper Preprocessing Standard Method 1.1
ACQ Method: D:\Methods\flexControlMethods\MBT_FC.par
ACQ Timestamp: 2025-02-26T12:37:35.502
AutoXecute Method: MBT_AutoX
Applied Taxonomy Tree: Taxonomy, Bruker Taxonomy, Projects, Sajat izolatumok

Escherichia coli	10 hits	2.43	2.36	2.32	2.31	2.28	2.25	2.21	2.16	2.15	2.14

Sample 12

Sample Name: F11
Sample Description:
Sample ID: 52
Sample Creation Date/Time: 2025-02-26T12:30:42.453
Sample Type: Standard
Identification Method: MALDI Biotyper MSP Identification Standard Method 1.1
Preprocessing Method: MALDI Biotyper Preprocessing Standard Method 1.1
ACQ Method: D:\Methods\flexControlMethods\MBT_FC.par
ACQ Timestamp: 2025-02-26T12:38:08.825
AutoXecute Method: MBT_AutoX
Applied Taxonomy Tree: Projects, Sajat izolatumok, Bruker Taxonomy, Taxonomy

Escherichia coli	10 hits	2.36	2.33	2.30	2.30	2.23	2.22	2.13	2.09	2.09	2.08

Sample 13

Sample Name: F12
Sample Description:
Sample ID: 52
Sample Creation Date/Time: 2025-02-26T12:30:42.456
Sample Type: Standard
Identification Method: MALDI Biotyper MSP Identification Standard Method 1.1
Preprocessing Method: MALDI Biotyper Preprocessing Standard Method 1.1
ACQ Method: D:\Methods\flexControlMethods\MBT_FC.par
ACQ Timestamp: 2025-02-26T12:38:52.033
AutoXecute Method: MBT_AutoX
Applied Taxonomy Tree: Projects, Bruker Taxonomy, Taxonomy, Sajat izolatumok

Paenibacillus provencensis	2 hits	2.24	2.07								
Escherichia coli	8 hits			1.97	1.95	1.92	1.90	1.89	1.88	1.81	1.78

Sample 14

Sample Name: G1
Sample Description:
Sample ID: 57
Sample Creation Date/Time: 2025-02-26T12:30:42.458
Sample Type: Standard
Identification Method: MALDI Biotyper MSP Identification Standard Method 1.1
Preprocessing Method: MALDI Biotyper Preprocessing Standard Method 1.1
ACQ Method: D:\Methods\flexControlMethods\MBT_FC.par
ACQ Timestamp: 2025-02-26T12:39:23.989
AutoXecute Method: MBT_AutoX
Applied Taxonomy Tree: Taxonomy, Bruker Taxonomy, Projects, Sajat izolatumok

Escherichia coli	9 hits	2.44	2.44	2.36	2.34	2.33	2.23	2.20	2.19	2.11	
Escherichia fergusonii	1 hit										2.08

Sample 15

Sample Name: G2
Sample Description:
Sample ID: 58
Sample Creation Date/Time: 2025-02-26T12:30:42.461
Sample Type: Standard
Identification Method: MALDI Biotyper MSP Identification Standard Method 1.1
Preprocessing Method: MALDI Biotyper Preprocessing Standard Method 1.1
ACQ Method: D:\Methods\flexControlMethods\MBT_FC.par
ACQ Timestamp: 2025-02-26T12:40:03.203
AutoXecute Method: MBT_AutoX
Applied Taxonomy Tree: Taxonomy, Sajat izolatumok, Projects, Bruker Taxonomy

Escherichia coli	10 hits	2.36	2.31	2.30	2.29	2.23	2.22	2.13	2.07	2.06	2.05

Sample 16

Sample Name: G3
Sample Description:
Sample ID: 62
Sample Creation Date/Time: 2025-02-26T12:30:42.463
Sample Type: Standard
Identification Method: MALDI Biotyper MSP Identification Standard Method 1.1
Preprocessing Method: MALDI Biotyper Preprocessing Standard Method 1.1
ACQ Method: D:\Methods\flexControlMethods\MBT_FC.par
ACQ Timestamp: 2025-02-26T12:40:42.192
AutoXecute Method: MBT_AutoX
Applied Taxonomy Tree: Taxonomy, Bruker Taxonomy, Projects, Sajat izolatumok

Escherichia coli	8 hits	2.20	2.18		2.09	2.09	2.07	2.06	2.05	2.02	
Enterobacter cloacae	1 hit			2.10							
Enterobacter hormaechei	1 hit									2.02	

Sample 17

Sample Name: G4
Sample Description:
Sample ID: 64
Sample Creation Date/Time: 2025-02-26T12:30:42.465
Sample Type: Standard
Identification Method: MALDI Biotyper MSP Identification Standard Method 1.1
Preprocessing Method: MALDI Biotyper Preprocessing Standard Method 1.1
ACQ Method: D:\Methods\flexControlMethods\MBT_FC.par
ACQ Timestamp: 2025-02-26T12:41:05.301
AutoXecute Method: MBT_AutoX
Applied Taxonomy Tree: Taxonomy, Bruker Taxonomy, Projects, Sajat izolatumok

Escherichia coli	10 hits	2.36	2.28	2.25	2.24	2.24	2.18	2.16	2.15	2.11	2.08

Sample 18

Sample Name: G5
Sample Description:
Sample ID: 64
Sample Creation Date/Time: 2025-02-26T12:30:42.468
Sample Type: Standard
Identification Method: MALDI Biotyper MSP Identification Standard Method 1.1
Preprocessing Method: MALDI Biotyper Preprocessing Standard Method 1.1
ACQ Method: D:\Methods\flexControlMethods\MBT_FC.par
ACQ Timestamp: 2025-02-26T12:41:39.660
AutoXecute Method: MBT_AutoX
Applied Taxonomy Tree: Sajat izolatumok, Projects, Bruker Taxonomy, Taxonomy

Escherichia coli	9 hits	2.49	2.39	2.36	2.31	2.27	2.26	2.20	2.16	2.15	
Escherichia fergusonii	1 hit										2.04

Matching Hints

Matched Pattern	Comment
Bacillus cereus CICC 23949 CICC	Bacillus anthracis, cereus, mycoides, pseudomycoides and thuringiensis are closely related and members of the Bacillus cereus group. In particular Bacillus cereus spectra are very similar to spectra from Bacillus anthracis. Bacillus anthracis is not included in the MALDI Biotyper database. For differentiation an adequate identification method has to be selected by an experienced professional. The quality of spectra (score) depends on the degree of sporulation: Use fresh material.
Bacillus cereus DSM 31T DSM	Bacillus anthracis, cereus, mycoides, pseudomycoides and thuringiensis are closely related and members of the Bacillus cereus group. In particular Bacillus cereus spectra are very similar to spectra from Bacillus anthracis. Bacillus anthracis is not included in the MALDI Biotyper database. For differentiation an adequate identification method has to be selected by an experienced professional. The quality of spectra (score) depends on the degree of sporulation: Use fresh material.
Bacillus cytotoxicus 1Z46778_1e MVD	The quality of spectra (score) depends on the degree of sporulation: Use fresh material.
Bacillus mycoides DSM 11821T DSM	Bacillus anthracis, cereus, mycoides, pseudomycoides and thuringiensis are closely related and members of the Bacillus cereus group. In particular Bacillus cereus spectra are very similar to spectra from Bacillus anthracis. Bacillus anthracis is not included in the MALDI Biotyper database. For differentiation an adequate identification method has to be selected by an experienced professional. The quality of spectra (score) depends on the degree of sporulation: Use fresh material.
Bacillus mycoides DSM 11821T DSM_2	Bacillus anthracis, cereus, mycoides, pseudomycoides and thuringiensis are closely related and members of the Bacillus cereus group. In particular Bacillus cereus spectra are very similar to spectra from Bacillus anthracis. Bacillus anthracis is not included in the MALDI Biotyper database. For differentiation an adequate identification method has to be selected by an experienced professional. The quality of spectra (score) depends on the degree of sporulation: Use fresh material.
Bacillus mycoides DSM 2048T DSM	Bacillus anthracis, cereus, mycoides, pseudomycoides and thuringiensis are closely related and members of the Bacillus cereus group. In particular Bacillus cereus spectra are very similar to spectra from Bacillus anthracis. Bacillus anthracis is not included in the MALDI Biotyper database. For differentiation an adequate identification method has to be selected by an experienced professional. The quality of spectra (score) depends on the degree of sporulation: Use fresh material.
Bacillus pseudomycoides DSM 12442T DSM	Bacillus anthracis, cereus, mycoides, pseudomycoides and thuringiensis are closely related and members of the Bacillus cereus group. In particular Bacillus cereus spectra are very similar to spectra from Bacillus anthracis. Bacillus anthracis is not included in the MALDI Biotyper database. For differentiation an adequate identification method has to be selected by an experienced professional. The quality of spectra (score) depends on the degree of sporulation: Use fresh material.
Bacillus thuringiensis DSM 2046T DSM	Bacillus anthracis, cereus, mycoides, pseudomycoides and thuringiensis are closely related and members of the Bacillus cereus group. In particular Bacillus cereus spectra are very similar to spectra from Bacillus anthracis. Bacillus anthracis is not included in the MALDI Biotyper database. For differentiation an adequate identification method has to be selected by an experienced professional. The quality of spectra (score) depends on the degree of sporulation: Use fresh material.

Matching Hints table--continued on next page

Matching Hints table--continued from previous page	
**Matched Pattern**	**Comment**
Enterobacter cloacae MB11506_1 CHB	is a member of Enterobacter cloacae complex
Enterobacter hormaechei ssp xiangfangensis DSM 101093T DSM	is a member of Enterobacter cloacae complex
Escherichia coli ATCC 25922 CHB	closely related to Shigella / Escherichia fergusonii and not definitely distinguishable at the moment
Escherichia coli ATCC 25922 THL	closely related to Shigella / Escherichia fergusonii and not definitely distinguishable at the moment
Escherichia coli ATCC 35218 CHB	closely related to Shigella / Escherichia fergusonii and not definitely distinguishable at the moment
Escherichia coli B421 UFL	closely related to Shigella / Escherichia fergusonii and not definitely distinguishable at the moment
Escherichia coli DH5alpha BRL	closely related to Shigella / Escherichia fergusonii and not definitely distinguishable at the moment
Escherichia coli DSM 1103_QC DSM	closely related to Shigella / Escherichia fergusonii and not definitely distinguishable at the moment
Escherichia coli DSM 1576 DSM	closely related to Shigella / Escherichia fergusonii and not definitely distinguishable at the moment
Escherichia coli DSM 30083T HAM	closely related to Shigella / Escherichia fergusonii and not definitely distinguishable at the moment
Escherichia coli DSM 682 DSM	closely related to Shigella / Escherichia fergusonii and not definitely distinguishable at the moment
Escherichia coli ESBL_EA_RSS_1528T CHB	closely related to Shigella / Escherichia fergusonii and not definitely distinguishable at the moment
Escherichia coli MB11464_1 CHB	closely related to Shigella / Escherichia fergusonii and not definitely distinguishable at the moment
Escherichia coli Nissl VML	closely related to Shigella / Escherichia fergusonii and not definitely distinguishable at the moment
Escherichia coli RV412_A1_2010_06a LBK	closely related to Shigella / Escherichia fergusonii and not definitely distinguishable at the moment
Escherichia coli W3350 MMG	closely related to Shigella / Escherichia fergusonii and not definitely distinguishable at the moment
Matching Hints table--continued on next page	

Matched Pattern	Comment
Matching Hints table--continued from previous page	
Escherichia fergusonii DSM 13698T HAM	closely related to Shigella / Escherichia coli and not definitely distinguishable at the moment
Neisseria meningitidis Serogroup_W135 BRL	Non-pathogenic Neisseria species could be misidentified as Neisseria meningitidis. For differentiation an adequate identification method has to be selected by an experienced professional.
Paenibacillus provencensis 190125_75777 RQCL	For the species provencensis / shunpengii / urinalis of the genus Paenibacillus the 16S rRNA gene sequences are very similar. Therefore distinguishing the mentioned species is difficult.
Paenibacillus provencensis CICC 23958 CICC	For the species provencensis / shunpengii / urinalis of the genus Paenibacillus the 16S rRNA gene sequences are very similar. Therefore distinguishing the mentioned species is difficult.

Meaning of Score Values

Range	Interpretation	Symbols	Color
2.00 - 3.00	High-confidence identification	(+++)	green
1.70 - 1.99	Low-confidence identification	(+)	yellow
0.00 - 1.69	No Organism Identification Possible	(-)	red

Meaning of Consistency Categories (A - C)

Category	Interpretation
(A)	**High consistency:** The best match is a high-confidence identification. The second-best match is (1) a high-confidence identification in which the species is identical to the best match, (2) a low-confidence identification in which the species or genus is identical to the best match, or (3) a non-identification.
(B)	**Low consistency:** The requirements for high consistency are not met. The best match is a high- or low-confidence identification. The second-best match is (1) a high- or low-confidence identification in which the genus is identical to the best match or (2) a non-identification.
(C)	**No consistency:** The requirements for high or low consistency are not met.

Sample 1

**Sample Name:**	E12
**Sample Description:**	
**Sample ID:**	1
**Sample Creation Date/Time:**	2025-02-26T12:30:42.427
**Sample Type:**	Standard
**Identification Method:**	MALDI Biotyper MSP Identification Standard Method 1.1
**Preprocessing Method:**	MALDI Biotyper Preprocessing Standard Method 1.1
**ACQ Method:**	D:\Methods\flexControlMethods\MBT_FC.par
**AutoXecute Method:**	MBT_AutoX
**Consistency Category (based on 2 best matches):**	A
**Applied Taxonomy Tree:**	Taxonomy, Bruker Taxonomy, Projects, Sajat izolatumok

Rank (Quality)	Matched Pattern	Score Value	NCBI Identifier
1 (+++)	Escherichia coli DH5alpha BRL	2.26	562
2 (+++)	Escherichia coli MB11464_1 CHB	2.23	562
3 (+++)	Escherichia coli DSM 682 DSM	2.20	562
4 (+++)	Escherichia coli RV412_A1_2010_06a LBK	2.20	562
5 (+++)	Escherichia coli ATCC 25922 THL	2.19	562
6 (+++)	Escherichia coli DSM 1576 DSM	2.12	562
7 (+++)	Escherichia coli ATCC 25922 CHB	2.05	562
8 (+++)	Escherichia coli Nissl VML	2.03	562
9 (+++)	Escherichia coli ATCC 35218 CHB	2.01	562
10 (+++)	Escherichia coli W3350 MMG	2.00	562

Sample 2

**Sample Name:**	F1
**Sample Description:**	
**Sample ID:**	2
**Sample Creation Date/Time:**	2025-02-26T12:30:42.429
**Sample Type:**	Standard
**Identification Method:**	MALDI Biotyper MSP Identification Standard Method 1.1
**Preprocessing Method:**	MALDI Biotyper Preprocessing Standard Method 1.1
**ACQ Method:**	D:\Methods\flexControlMethods\MBT_FC.par
**AutoXecute Method:**	MBT_AutoX
**Consistency Category (based on 2 best matches):**	A
**Applied Taxonomy Tree:**	Bruker Taxonomy, Taxonomy, Sajat izolatumok, Projects

Rank (Quality)	Matched Pattern	Score Value	NCBI Identifier
1 (+++)	Escherichia coli RV412_A1_2010_06a LBK	2.23	562
2 (+++)	Escherichia coli DSM 1576 DSM	2.22	562
3 (+++)	Escherichia coli DSM 682 DSM	2.19	562
4 (+++)	Escherichia coli MB11464_1 CHB	2.16	562
5 (+++)	Escherichia coli ATCC 25922 THL	2.16	562
6 (+++)	Escherichia coli DH5alpha BRL	2.07	562
7 (+++)	Escherichia coli Nissl VML	2.01	562
8 (+)	Escherichia coli ESBL_EA_RSS_1528T CHB	1.99	562
9 (+)	Escherichia coli ATCC 25922 CHB	1.97	562
10 (+)	Escherichia coli ATCC 35218 CHB	1.95	562

Sample 3

HUNGARIAN UNIVERSITY OF AGRICULTURE AND LIFE SCIENCES

**Sample Name:**	F2
**Sample Description:**	
**Sample ID:**	3
**Sample Creation Date/Time:**	2025-02-26T12:30:42.431
**Sample Type:**	Standard
**Identification Method:**	MALDI Biotyper MSP Identification Standard Method 1.1
**Preprocessing Method:**	MALDI Biotyper Preprocessing Standard Method 1.1
**ACQ Method:**	D:\Methods\flexControlMethods\MBT_FC.par
**AutoXecute Method:**	MBT_AutoX
**Consistency Category (based on 2 best matches):**	A
**Applied Taxonomy Tree:**	Sajat izolatumok, Projects, Bruker Taxonomy, Taxonomy

Rank (Quality)	Matched Pattern	Score Value	NCBI Identifier
1 (+++)	Escherichia coli DSM 682 DSM	2.30	562
2 (+++)	Escherichia coli RV412_A1_2010_06a LBK	2.28	562
3 (+++)	Escherichia coli ATCC 25922 THL	2.26	562
4 (+++)	Escherichia coli MB11464_1 CHB	2.22	562
5 (+++)	Escherichia coli DH5alpha BRL	2.20	562
6 (+++)	Escherichia coli DSM 1576 DSM	2.20	562
7 (+++)	Escherichia coli ATCC 25922 CHB	2.06	562
8 (+++)	Escherichia coli ESBL_EA_RSS_1528T CHB	2.05	562
9 (+++)	Escherichia coli ATCC 35218 CHB	2.03	562
10 (+)	Escherichia coli Nissl VML	1.96	562

Sample 4

HUNGARIAN UNIVERSITY OF AGRICULTURE AND LIFE SCIENCES

**Sample Name:**	F3
**Sample Description:**	
**Sample ID:**	7
**Sample Creation Date/Time:**	2025-02-26T12:30:42.434
**Sample Type:**	Standard
**Identification Method:**	MALDI Biotyper MSP Identification Standard Method 1.1
**Preprocessing Method:**	MALDI Biotyper Preprocessing Standard Method 1.1
**ACQ Method:**	D:\Methods\flexControlMethods\MBT_FC.par
**AutoXecute Method:**	MBT_AutoX
**Consistency Category (based on 2 best matches):**	A
**Applied Taxonomy Tree:**	Sajat izolatumok, Projects, Bruker Taxonomy, Taxonomy

Rank (Quality)	Matched Pattern	Score Value	NCBI Identifier
1 (+++)	Escherichia coli DSM 682 DSM	2.44	562
2 (+++)	Escherichia coli DSM 1576 DSM	2.41	562
3 (+++)	Escherichia coli RV412_A1_2010_06a LBK	2.31	562
4 (+++)	Escherichia coli ATCC 25922 THL	2.28	562
5 (+++)	Escherichia coli MB11464_1 CHB	2.28	562
6 (+++)	Escherichia coli DSM 1103_QC DSM	2.18	562
7 (+++)	Escherichia coli ATCC 25922 CHB	2.18	562
8 (+++)	Escherichia coli DH5alpha BRL	2.17	562
9 (+++)	Escherichia coli Nissl VML	2.16	562
10 (+++)	Escherichia coli ESBL_EA_RSS_1528T CHB	2.13	562

Sample 5

MATE
HUNGARIAN UNIVERSITY OF AGRICULTURE AND LIFE SCIENCES

**Sample Name:**	F4
**Sample Description:**	
**Sample ID:**	8
**Sample Creation Date/Time:**	2025-02-26T12:30:42.436
**Sample Type:**	Standard
**Identification Method:**	MALDI Biotyper MSP Identification Standard Method 1.1
**Preprocessing Method:**	MALDI Biotyper Preprocessing Standard Method 1.1
**ACQ Method:**	D:\Methods\flexControlMethods\MBT_FC.par
**AutoXecute Method:**	MBT_AutoX
**Consistency Category (based on 2 best matches):**	A
**Applied Taxonomy Tree:**	Taxonomy, Bruker Taxonomy, Projects, Sajat izolatumok

Rank (Quality)	Matched Pattern	Score Value	NCBI Identifier
1 (+++)	Escherichia coli RV412_A1_2010_06a LBK	2.46	562
2 (+++)	Escherichia coli DSM 1576 DSM	2.45	562
3 (+++)	Escherichia coli DSM 682 DSM	2.41	562
4 (+++)	Escherichia coli MB11464_1 CHB	2.33	562
5 (+++)	Escherichia coli ATCC 25922 THL	2.29	562
6 (+++)	Escherichia coli DH5alpha BRL	2.23	562
7 (+++)	Escherichia coli DSM 1103_QC DSM	2.20	562
8 (+++)	Escherichia coli Nissl VML	2.13	562
9 (+++)	Escherichia coli ATCC 25922 CHB	2.11	562
10 (+++)	Escherichia coli ESBL_EA_RSS_1528T CHB	2.10	562

Sample 6

HUNGARIAN UNIVERSITY OF AGRICULTURE AND LIFE SCIENCES

**Sample Name:**	F5
**Sample Description:**	
**Sample ID:**	16
**Sample Creation Date/Time:**	2025-02-26T12:30:42.438
**Sample Type:**	Standard
**Identification Method:**	MALDI Biotyper MSP Identification Standard Method 1.1
**Preprocessing Method:**	MALDI Biotyper Preprocessing Standard Method 1.1
**ACQ Method:**	D:\Methods\flexControlMethods\MBT_FC.par
**AutoXecute Method:**	MBT_AutoX
**Consistency Category (based on 2 best matches):**	A
**Applied Taxonomy Tree:**	Projects, Sajat izolatumok, Taxonomy, Bruker Taxonomy

Rank (Quality)	Matched Pattern	Score Value	NCBI Identifier
1 (+++)	Bacillus thuringiensis DSM 2046T DSM	2.03	1428
2 (+)	Bacillus mycoides DSM 2048T DSM	1.95	1405
3 (+)	Bacillus mycoides DSM 11821T DSM	1.92	1405
4 (+)	Bacillus cereus DSM 31T DSM	1.92	1396
5 (+)	Bacillus mycoides DSM 11821T DSM_2	1.88	1405
6 (+)	Bacillus cereus CICC 23949 CICC	1.87	1396
7 (-)	Bacillus pseudomycoides DSM 12442T DSM	1.65	64104
8 (-)	Acinetobacter towneri DSM 14962T HAM	1.55	202956
9 (-)	Bacillus cytotoxicus 1Z46778_1e MVD	1.51	1386
10 (-)	Neisseria meningitidis Serogroup_W135 BRL	1.50	487

Sample 7

HUNGARIAN UNIVERSITY OF AGRICULTURE AND LIFE SCIENCES

**Sample Name:**	F6
**Sample Description:**	
**Sample ID:**	25
**Sample Creation Date/Time:**	2025-02-26T12:30:42.441
**Sample Type:**	Standard
**Identification Method:**	MALDI Biotyper MSP Identification Standard Method 1.1
**Preprocessing Method:**	MALDI Biotyper Preprocessing Standard Method 1.1
**ACQ Method:**	D:\Methods\flexControlMethods\MBT_FC.par
**AutoXecute Method:**	MBT_AutoX
**Consistency Category (based on 2 best matches):**	A
**Applied Taxonomy Tree:**	Projects, Bruker Taxonomy, Sajat izolatumok, Taxonomy

Rank (Quality)	Matched Pattern	Score Value	NCBI Identifier
1 (+++)	Escherichia coli DSM 1576 DSM	2.52	562
2 (+++)	Escherichia coli DSM 682 DSM	2.35	562
3 (+++)	Escherichia coli ATCC 25922 THL	2.23	562
4 (+++)	Escherichia coli RV412_A1_2010_06a LBK	2.22	562
5 (+++)	Escherichia coli MB11464_1 CHB	2.21	562
6 (+++)	Escherichia coli ATCC 25922 CHB	2.16	562
7 (+++)	Escherichia coli ESBL_EA_RSS_1528T CHB	2.12	562
8 (+++)	Escherichia coli DSM 1103_QC DSM	2.09	562
9 (+++)	Escherichia coli Nissl VML	2.08	562
10 (+++)	Escherichia coli B421 UFL	2.04	562

Sample 8

HUNGARIAN UNIVERSITY OF AGRICULTURE AND LIFE SCIENCES

**Sample Name:**	F7
**Sample Description:**	
**Sample ID:**	26
**Sample Creation Date/Time:**	2025-02-26T12:30:42.443
**Sample Type:**	Standard
**Identification Method:**	MALDI Biotyper MSP Identification Standard Method 1.1
**Preprocessing Method:**	MALDI Biotyper Preprocessing Standard Method 1.1
**ACQ Method:**	D:\Methods\flexControlMethods\MBT_FC.par
**AutoXecute Method:**	MBT_AutoX
**Consistency Category (based on 2 best matches):**	A
**Applied Taxonomy Tree:**	Sajat izolatumok, Projects, Bruker Taxonomy, Taxonomy

Rank (Quality)	Matched Pattern	Score Value	NCBI Identifier
1 (+++)	Escherichia coli DSM 1576 DSM	2.51	562
2 (+++)	Escherichia coli RV412_A1_2010_06a LBK	2.38	562
3 (+++)	Escherichia coli DSM 682 DSM	2.34	562
4 (+++)	Escherichia coli ATCC 25922 THL	2.27	562
5 (+++)	Escherichia coli MB11464_1 CHB	2.26	562
6 (+++)	Escherichia coli ESBL_EA_RSS_1528T CHB	2.25	562
7 (+++)	Escherichia coli DH5alpha BRL	2.22	562
8 (+++)	Escherichia coli DSM 1103_QC DSM	2.20	562
9 (+++)	Escherichia coli ATCC 25922 CHB	2.19	562
10 (+++)	Escherichia coli Nissl VML	2.16	562

Sample 9

HUNGARIAN UNIVERSITY OF AGRICULTURE AND LIFE SCIENCES

**Sample Name:**	F8
**Sample Description:**	
**Sample ID:**	29
**Sample Creation Date/Time:**	2025-02-26T12:30:42.446
**Sample Type:**	Standard
**Identification Method:**	MALDI Biotyper MSP Identification Standard Method 1.1
**Preprocessing Method:**	MALDI Biotyper Preprocessing Standard Method 1.1
**ACQ Method:**	D:\Methods\flexControlMethods\MBT_FC.par
**AutoXecute Method:**	MBT_AutoX
**Consistency Category (based on 2 best matches):**	A
**Applied Taxonomy Tree:**	Sajat izolatumok, Bruker Taxonomy, Projects, Taxonomy

Rank (Quality)	Matched Pattern	Score Value	NCBI Identifier
1 (+++)	Escherichia coli DSM 1576 DSM	2.44	562
2 (+++)	Escherichia coli DSM 682 DSM	2.40	562
3 (+++)	Escherichia coli RV412_A1_2010_06a LBK	2.36	562
4 (+++)	Escherichia coli ATCC 25922 THL	2.30	562
5 (+++)	Escherichia coli MB11464_1 CHB	2.26	562
6 (+++)	Escherichia coli DH5alpha BRL	2.26	562
7 (+++)	Escherichia coli ESBL_EA_RSS_1528T CHB	2.17	562
8 (+++)	Escherichia coli DSM 1103_QC DSM	2.16	562
9 (+++)	Escherichia coli Nissl VML	2.14	562
10 (+++)	Escherichia coli ATCC 25922 CHB	2.12	562

Sample 10

HUNGARIAN UNIVERSITY OF AGRICULTURE AND LIFE SCIENCES

**Sample Name:**	F9
**Sample Description:**	
**Sample ID:**	47
**Sample Creation Date/Time:**	2025-02-26T12:30:42.448
**Sample Type:**	Standard
**Identification Method:**	MALDI Biotyper MSP Identification Standard Method 1.1
**Preprocessing Method:**	MALDI Biotyper Preprocessing Standard Method 1.1
**ACQ Method:**	D:\Methods\flexControlMethods\MBT_FC.par
**AutoXecute Method:**	MBT_AutoX
**Consistency Category (based on 2 best matches):**	A
**Applied Taxonomy Tree:**	Sajat izolatumok, Projects, Bruker Taxonomy, Taxonomy

Rank (Quality)	Matched Pattern	Score Value	NCBI Identifier
1 (+++)	Escherichia coli DH5alpha BRL	2.36	562
2 (+++)	Escherichia coli DSM 682 DSM	2.25	562
3 (+++)	Escherichia coli DSM 1576 DSM	2.24	562
4 (+++)	Escherichia coli ATCC 25922 THL	2.23	562
5 (+++)	Escherichia coli RV412_A1_2010_06a LBK	2.23	562
6 (+++)	Escherichia coli MB11464_1 CHB	2.13	562
7 (+++)	Escherichia coli W3350 MMG	2.10	562
8 (+++)	Escherichia coli ESBL_EA_RSS_1528T CHB	2.09	562
9 (+++)	Escherichia coli DSM 30083T HAM	2.08	562
10 (+++)	Escherichia coli DSM 1103_QC DSM	2.08	562

Sample 11

**Sample Name:**	F10
**Sample Description:**	
**Sample ID:**	49
**Sample Creation Date/Time:**	2025-02-26T12:30:42.451
**Sample Type:**	Standard
**Identification Method:**	MALDI Biotyper MSP Identification Standard Method 1.1
**Preprocessing Method:**	MALDI Biotyper Preprocessing Standard Method 1.1
**ACQ Method:**	D:\Methods\flexControlMethods\MBT_FC.par
**AutoXecute Method:**	MBT_AutoX
**Consistency Category (based on 2 best matches):**	A
**Applied Taxonomy Tree:**	Taxonomy, Bruker Taxonomy, Projects, Sajat izolatumok

Rank (Quality)	Matched Pattern	Score Value	NCBI Identifier
1 (+++)	Escherichia coli DSM 682 DSM	2.43	562
2 (+++)	Escherichia coli DSM 1576 DSM	2.36	562
3 (+++)	Escherichia coli DH5alpha BRL	2.32	562
4 (+++)	Escherichia coli ATCC 25922 THL	2.31	562
5 (+++)	Escherichia coli MB11464_1 CHB	2.28	562
6 (+++)	Escherichia coli DSM 1103_QC DSM	2.25	562
7 (+++)	Escherichia coli RV412_A1_2010_06a LBK	2.21	562
8 (+++)	Escherichia coli ATCC 25922 CHB	2.16	562
9 (+++)	Escherichia coli Nissl VML	2.15	562
10 (+++)	Escherichia coli ESBL_EA_RSS_1528T CHB	2.14	562

Sample 12

**Sample Name:**	F11
**Sample Description:**	
**Sample ID:**	52
**Sample Creation Date/Time:**	2025-02-26T12:30:42.453
**Sample Type:**	Standard
**Identification Method:**	MALDI Biotyper MSP Identification Standard Method 1.1
**Preprocessing Method:**	MALDI Biotyper Preprocessing Standard Method 1.1
**ACQ Method:**	D:\Methods\flexControlMethods\MBT_FC.par
**AutoXecute Method:**	MBT_AutoX
**Consistency Category (based on 2 best matches):**	C
**Applied Taxonomy Tree:**	Projects, Sajat izolatumok, Bruker Taxonomy, Taxonomy

Rank (Quality)	Matched Pattern	Score Value	NCBI Identifier
1 (+++)	Escherichia coli DSM 682 DSM	2.36	562
2 (+++)	Escherichia coli DSM 1576 DSM	2.33	562
3 (+++)	Escherichia coli RV412_A1_2010_06a LBK	2.30	562
4 (+++)	Escherichia coli MB11464_1 CHB	2.30	562
5 (+++)	Escherichia coli DH5alpha BRL	2.23	562
6 (+++)	Escherichia coli ATCC 25922 THL	2.22	562
7 (+++)	Escherichia coli ESBL_EA_RSS_1528T CHB	2.13	562
8 (+++)	Escherichia coli Nissl VML	2.09	562
9 (+++)	Escherichia coli ATCC 25922 CHB	2.09	562
10 (+++)	Escherichia coli DSM 1103_QC DSM	2.08	562

Sample 13

HUNGARIAN UNIVERSITY OF AGRICULTURE AND LIFE SCIENCES

**Sample Name:**	F12
**Sample Description:**	
**Sample ID:**	52
**Sample Creation Date/Time:**	2025-02-26T12:30:42.456
**Sample Type:**	Standard
**Identification Method:**	MALDI Biotyper MSP Identification Standard Method 1.1
**Preprocessing Method:**	MALDI Biotyper Preprocessing Standard Method 1.1
**ACQ Method:**	D:\Methods\flexControlMethods\MBT_FC.par
**AutoXecute Method:**	MBT_AutoX
**Consistency Category (based on 2 best matches):**	C
**Applied Taxonomy Tree:**	Projects, Bruker Taxonomy, Taxonomy, Sajat izolatumok

Rank (Quality)	Matched Pattern	Score Value	NCBI Identifier
1 (+++)	Paenibacillus provencensis CICC 23958 CICC	2.24	44249
2 (+++)	Paenibacillus provencensis 190125_75777 RQCL	2.07	44249
3 (+)	Escherichia coli ATCC 25922 THL	1.97	562
4 (+)	Escherichia coli DH5alpha BRL	1.95	562
5 (+)	Escherichia coli DSM 682 DSM	1.92	562
6 (+)	Escherichia coli MB11464_1 CHB	1.90	562
7 (+)	Escherichia coli RV412_A1_2010_06a LBK	1.89	562
8 (+)	Escherichia coli DSM 1576 DSM	1.88	562
9 (+)	Escherichia coli DSM 1103_QC DSM	1.81	562
10 (+)	Escherichia coli ATCC 25922 CHB	1.78	562

Sample 14

**Sample Name:**	G1
**Sample Description:**	
**Sample ID:**	57
**Sample Creation Date/Time:**	2025-02-26T12:30:42.458
**Sample Type:**	Standard
**Identification Method:**	MALDI Biotyper MSP Identification Standard Method 1.1
**Preprocessing Method:**	MALDI Biotyper Preprocessing Standard Method 1.1
**ACQ Method:**	D:\Methods\flexControlMethods\MBT_FC.par
**AutoXecute Method:**	MBT_AutoX
**Consistency Category (based on 2 best matches):**	A
**Applied Taxonomy Tree:**	Taxonomy, Bruker Taxonomy, Projects, Sajat izolatumok

Rank (Quality)	Matched Pattern	Score Value	NCBI Identifier
1 (+++)	Escherichia coli DSM 1576 DSM	2.44	562
2 (+++)	Escherichia coli DSM 682 DSM	2.44	562
3 (+++)	Escherichia coli RV412_A1_2010_06a LBK	2.36	562
4 (+++)	Escherichia coli ATCC 25922 THL	2.34	562
5 (+++)	Escherichia coli MB11464_1 CHB	2.33	562
6 (+++)	Escherichia coli DH5alpha BRL	2.23	562
7 (+++)	Escherichia coli DSM 1103_QC DSM	2.20	562
8 (+++)	Escherichia coli Nissl VML	2.19	562
9 (+++)	Escherichia coli ATCC 25922 CHB	2.11	562
10 (+++)	Escherichia fergusonii DSM 13698T HAM	2.08	564

Sample 15

HUNGARIAN UNIVERSITY OF AGRICULTURE AND LIFE SCIENCES

**Sample Name:**	G2
**Sample Description:**	
**Sample ID:**	58
**Sample Creation Date/Time:**	2025-02-26T12:30:42.461
**Sample Type:**	Standard
**Identification Method:**	MALDI Biotyper MSP Identification Standard Method 1.1
**Preprocessing Method:**	MALDI Biotyper Preprocessing Standard Method 1.1
**ACQ Method:**	D:\Methods\flexControlMethods\MBT_FC.par
**AutoXecute Method:**	MBT_AutoX
**Consistency Category (based on 2 best matches):**	A
**Applied Taxonomy Tree:**	Taxonomy, Sajat izolatumok, Projects, Bruker Taxonomy

Rank (Quality)	Matched Pattern	Score Value	NCBI Identifier
1 (+++)	Escherichia coli DSM 682 DSM	2.36	562
2 (+++)	Escherichia coli RV412_A1_2010_06a LBK	2.31	562
3 (+++)	Escherichia coli DH5alpha BRL	2.30	562
4 (+++)	Escherichia coli ATCC 25922 THL	2.29	562
5 (+++)	Escherichia coli DSM 1576 DSM	2.23	562
6 (+++)	Escherichia coli MB11464_1 CHB	2.22	562
7 (+++)	Escherichia coli Nissl VML	2.13	562
8 (+++)	Escherichia coli ATCC 25922 CHB	2.07	562
9 (+++)	Escherichia coli DSM 30083T HAM	2.06	562
10 (+++)	Escherichia coli ESBL_EA_RSS_1528T CHB	2.05	562

Sample 16

MATE HUNGARIAN UNIVERSITY OF AGRICULTURE AND LIFE SCIENCES

**Sample Name:**	G3
**Sample Description:**	
**Sample ID:**	62
**Sample Creation Date/Time:**	2025-02-26T12:30:42.463
**Sample Type:**	Standard
**Identification Method:**	MALDI Biotyper MSP Identification Standard Method 1.1
**Preprocessing Method:**	MALDI Biotyper Preprocessing Standard Method 1.1
**ACQ Method:**	D:\Methods\flexControlMethods\MBT_FC.par
**AutoXecute Method:**	MBT_AutoX
**Consistency Category (based on 2 best matches):**	A
**Applied Taxonomy Tree:**	Taxonomy, Bruker Taxonomy, Projects, Sajat izolatumok

Rank (Quality)	Matched Pattern	Score Value	NCBI Identifier
1 (+++)	Escherichia coli ATCC 25922 THL	2.20	562
2 (+++)	Escherichia coli DH5alpha BRL	2.18	562
3 (+++)	Enterobacter cloacae MB11506_1 CHB	2.10	550
4 (+++)	Escherichia coli DSM 1576 DSM	2.09	562
5 (+++)	Escherichia coli MB11464_1 CHB	2.09	562
6 (+++)	Escherichia coli ESBL_EA_RSS_1528T CHB	2.07	562
7 (+++)	Escherichia coli ATCC 25922 CHB	2.06	562
8 (+++)	Escherichia coli DSM 682 DSM	2.05	562
9 (+++)	Escherichia coli RV412_A1_2010_06a LBK	2.02	562
10 (+++)	Enterobacter hormaechei ssp xiangfangensis DSM 101093T DSM	2.02	158836

Sample 17

HUNGARIAN UNIVERSITY OF AGRICULTURE AND LIFE SCIENCES

**Sample Name:**	G4
**Sample Description:**	
**Sample ID:**	64
**Sample Creation Date/Time:**	2025-02-26T12:30:42.465
**Sample Type:**	Standard
**Identification Method:**	MALDI Biotyper MSP Identification Standard Method 1.1
**Preprocessing Method:**	MALDI Biotyper Preprocessing Standard Method 1.1
**ACQ Method:**	D:\Methods\flexControlMethods\MBT_FC.par
**AutoXecute Method:**	MBT_AutoX
**Consistency Category (based on 2 best matches):**	A
**Applied Taxonomy Tree:**	Taxonomy, Bruker Taxonomy, Projects, Sajat izolatumok

Rank (Quality)	Matched Pattern	Score Value	NCBI Identifier
1 (+++)	Escherichia coli DSM 682 DSM	2.36	562
2 (+++)	Escherichia coli MB11464_1 CHB	2.28	562
3 (+++)	Escherichia coli DSM 1103_QC DSM	2.25	562
4 (+++)	Escherichia coli ATCC 25922 THL	2.24	562
5 (+++)	Escherichia coli ATCC 25922 CHB	2.24	562
6 (+++)	Escherichia coli DH5alpha BRL	2.18	562
7 (+++)	Escherichia coli DSM 1576 DSM	2.16	562
8 (+++)	Escherichia coli ESBL_EA_RSS_1528T CHB	2.15	562
9 (+++)	Escherichia coli Nissl VML	2.11	562
10 (+++)	Escherichia coli RV412_A1_2010_06a LBK	2.08	562

Sample 18

**Sample Name:**	G5
**Sample Description:**	
**Sample ID:**	64
**Sample Creation Date/Time:**	2025-02-26T12:30:42.468
**Sample Type:**	Standard
**Identification Method:**	MALDI Biotyper MSP Identification Standard Method 1.1
**Preprocessing Method:**	MALDI Biotyper Preprocessing Standard Method 1.1
**ACQ Method:**	D:\Methods\flexControlMethods\MBT_FC.par
**AutoXecute Method:**	MBT_AutoX
**Consistency Category (based on 2 best matches):**	A
**Applied Taxonomy Tree:**	Sajat izolatumok, Projects, Bruker Taxonomy, Taxonomy

Rank (Quality)	Matched Pattern	Score Value	NCBI Identifier
1 (+++)	Escherichia coli DSM 682 DSM	2.49	562
2 (+++)	Escherichia coli DSM 1576 DSM	2.39	562
3 (+++)	Escherichia coli RV412_A1_2010_06a LBK	2.36	562
4 (+++)	Escherichia coli DSM 1103_QC DSM	2.31	562
5 (+++)	Escherichia coli ATCC 25922 THL	2.27	562
6 (+++)	Escherichia coli MB11464_1 CHB	2.26	562
7 (+++)	Escherichia coli ATCC 25922 CHB	2.20	562
8 (+++)	Escherichia coli DH5alpha BRL	2.16	562
9 (+++)	Escherichia coli ESBL_EA_RSS_1528T CHB	2.15	562
10 (+++)	Escherichia fergusonii DSM 13698T HAM	2.04	564
