# Supplementary material for: Virulence and Antimicrobial Resistance Traits of Escherichia coli Retrieved from Fermented Dairy Products During Ramadan in Egypt: Seasonal Public Health Implications
Source: Antibiotics (Basel). 2026 May 9;15(5):483. doi: 10.3390/antibiotics15050483 (PMC13203909; doi:10.3390/antibiotics15050483)
Supplement: Supplementary file 1 [file antibiotics-15-00483-s001.zip › Supplementary Table S1.pdf]

Supplementary Table S1

Antimicrobial resistance profiles, serotypes, and multiple antibiotic resistance (MAR) indices of *E.coli* isolates (n = 34) recovered from fermented dairy products (rayeb milk and yogurt). The table summarizes, for each isolate, the number of antibiotics (out of 16 tested) and antimicrobial categories (n = 10) to which resistance was observed, along with classification into multidrug-resistant (MDR) or extensively drug-resistant (XDR) phenotypes. The MAR index was calculated as the ratio of the number of antibiotics to which the isolate was resistant to the total number of antibiotics tested.

Isolate ID	Sample Origin	Serotype	No. of Resistant Antibiotics (n = 16)	No. of Resistant Categories (n = 10)	Resistance Classification	MAR Index
**19.0**	Rayeb Milk	O26: H11	7.0	5.0	MDR	0.4375
**56.0**	Rayeb Milk	O26: H11	9.0	6.0	MDR	0.5625
**22.0**	Rayeb Milk	O119: H6	10.0	8.0	XDR	0.625
**49.0**	Rayeb Milk	O153: H2	5.0	4.0	MDR	0.3125
**52.0**	Rayeb Milk	O153: H2	13.0	9.0	XDR	0.8125
**21.0**	Rayeb Milk	O153: H2	5.0	5.0	MDR	0.3125
**57.0**	Rayeb Milk	O26: H11	6.0	5.0	MDR	0.375
**69.0**	Rayeb Milk	O26: H11	5.0	4.0	MDR	0.3125
**16.0**	Rayeb Milk	O127: H6	10.0	7.0	MDR	0.625
**40.0**	Rayeb Milk	O127: H6	8.0	6.0	MDR	0.5
**51.0**	Rayeb Milk	O127: H6	9.0	7.0	MDR	0.5625
**97.0**	Yogurt	O119: H6	4.0	4.0	MDR	0.25
**76.0**	Yogurt	O119: H6	10.0	8.0	XDR	0.625
**83.0**	Yogurt	O119: H6	10.0	9.0	XDR	0.625
**103.0**	Yogurt	O119: H6	12.0	9.0	XDR	0.75
**109.0**	Yogurt	O111: H2	9.0	7.0	MDR	0.5625
**124.0**	Yogurt	O111: H2	7.0	6.0	MDR	0.4375
**138.0**	Yogurt	O111: H2	10.0	8.0	XDR	0.625
**85.0**	Yogurt	O111: H2	8.0	6.0	MDR	0.5
**117.0**	Yogurt	O125: H21	10.0	7.0	MDR	0.625
**150.0**	Yogurt	O125: H21	9.0	6.0	MDR	0.5625
**144.0**	Yogurt	O125: H21	8.0	6.0	MDR	0.5
**90.0**	Yogurt	O125: H21	10.0	7.0	MDR	0.625
**127.0**	Yogurt	O125: H21	8.0	5.0	MDR	0.5
**115.0**	Yogurt	O103: H2	6.0	6.0	MDR	0.375
**129.0**	Yogurt	O103: H2	6.0	6.0	MDR	0.375
**88.0**	Yogurt	O103: H2	6.0	6.0	MDR	0.375
**132.0**	Yogurt	O55: H7	11.0	8.0	XDR	0.6875
**101.0**	Yogurt	O55: H7	10.0	7.0	MDR	0.625
**92.0**	Yogurt	O127: H6	10.0	9.0	XDR	0.625
**131.0**	Yogurt	O153:H2	7.0	6.0	MDR	0.4375
**147.0**	Yogurt	O153:H2	7.0	6.0	MDR	0.4375
**108.0**	Yogurt	O153: H2	5.0	5.0	MDR	0.3125
**95.0**	Yogurt	O153:H2	9.0	8.0	XDR	0.5625
